# PROX1 loss in adult mouse Schlemm’s canal causes permanent ocular hypertension

**DOI:** 10.1172/jci.insight.203711

**Published:** 2026-05-05

**Authors:** Sofia Lara Ochoa, Hoi-Lam Li, Hyeohn Kim, Zihang Yan, Natalia C. Mendonca, Pan Liu, Hyunjoo J. Lee, Michael P. Vincent, Sultan Almunif, Hao F. Zhang, Haiyan Gong, Evan A. Scott, Mark Johnson, Benjamin R. Thomson

**Affiliations:** 1 Department of Biomedical Engineering, Northwestern University, Evanston, Illinois, USA.; 2 Department of Ophthalmology and Department of Anatomy and Neurobiology, Boston University School of Medicine, Boston, Massachusetts, USA.; 3 Section of Nephrology and Hypertension, Northwestern University Feinberg School of Medicine, Chicago, Illinois, USA.; 4 Massachusetts Eye and Ear, Boston, and Harvard Medical School, Cambridge, Massachusetts, USA.; 5 Department of Ophthalmology, Northwestern University Feinberg School of Medicine, Chicago, Illinois, USA.; 6 Department of Biomedical Engineering, NanoSTAR Institute, University of Virginia School of Medicine, Charlottesville, Virginia, USA.; 7 Feinberg Cardiovascular and Renal Research Institute, Northwestern University Feinberg School of Medicine, Chicago, Illinois, USA.

**Keywords:** Ophthalmology, Vascular biology, Endothelial cells, Mouse models, Nanotechnology

## Abstract

Glaucoma is associated with ocular hypertension, and lowering intraocular pressure is the primary objective of current therapies. Recent studies have established a key role for Schlemm’s canal endothelium in this pressure increase and have shown that it has a unique, lymphatic-like hybrid phenotype characterized by expression of the lymphatic transcription factor PROX1. However, the functional importance of this hybrid phenotype in the adult canal remains unclear, as long-term studies have been limited by systemic requirements for lymphatic gene expression and a lack of Schlemm’s canal–specific animal models. Here, we designed and validated a strategy using 4OH-tamoxifen-loaded nanocarriers to generate targeted, Schlemm’s canal specific *Prox1* knockout mice that specifically lacked lymphatic characteristics in the canal endothelium. Within 4 weeks, intraocular pressure was significantly elevated, and ocular hypertension was maintained for at least 24 weeks. Unlike lymphatic vessels, which degenerate following *Prox1* deletion, Schlemm’s canal persisted but reverted to a less functional vein-like phenotype with no change in size or morphology. Together, these findings demonstrate the utility of nanocarrier-mediated tamoxifen delivery and establish the importance of the Schlemm’s canal lymphatic-like phenotype in intraocular pressure regulation, providing targets for future glaucoma therapies and a mouse model of adult-onset ocular hypertension.

## Abstract 2

Nanocarrier-mediated, specific Prox1 deletion from the adult mouse Schlemm’s canal led to increased intraocular pressure, demonstrating the canal’s hybrid lymphatic-like identity is essential for outflow and intraocular pressure regulation.

## Graphical abstract

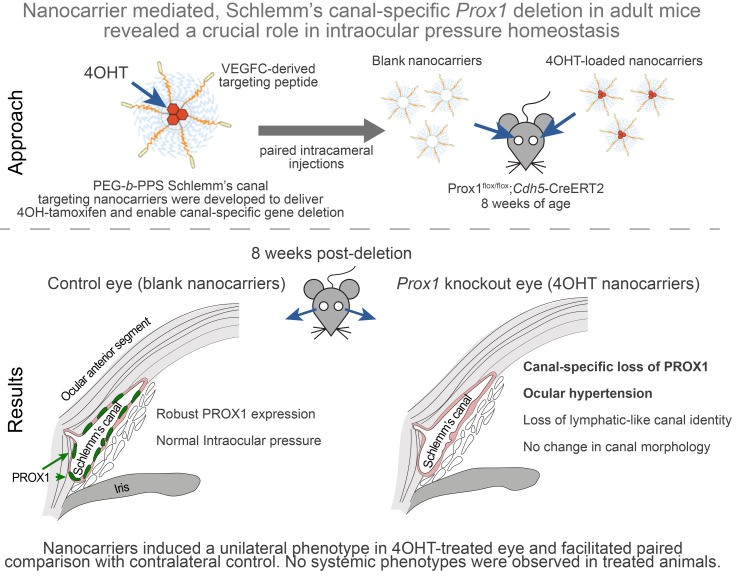

## Introduction

Glaucoma is a leading cause of vision loss worldwide, affecting an estimated 64 million people and leaving 3.6 million blind (1). While death of retinal ganglion cells is directly responsible for the vision loss, elevated intraocular pressure (IOP) is the only modifiable risk factor for development and progression of primary open-angle glaucoma, the most prevalent form of the disease. Pharmacological IOP reduction slows progression and lowers glaucoma risk (2, 3). However, even on maximal medical therapy, progressive visual field loss still occurs in some patients, indicating that current pharmacological treatments are not always sufficient and highlighting the need for new therapeutic approaches (4, 5). This is likely due to (a) the inability of current therapeutics to continuously keep IOP low enough to protect the optic nerve and (b) the lack of treatments that directly address the underlying pathology causing ocular hypertension.

The aqueous humor flows through the anterior segment, providing nutrition and removing metabolic wastes before leaving the eye primarily through an outflow pathway comprised of the trabecular meshwork and Schlemm’s canal—a unique vessel adjacent to the iridocorneal angle—and emptying into the collector channels and aqueous veins (6, 7). Resistance to flow through this pathway generates IOP, and in glaucoma, this flow resistance is elevated, causing ocular hypertension. A significant fraction of the total resistance is generated as aqueous humor passes through the deeper regions of the trabecular meshwork and/or the basal lamina of Schlemm’s canal before entering the canal through pores in its endothelial inner wall. These pores modulate outflow resistance (8), and their density is reduced in glaucoma (9, 10). It has recently been reported that this decreased pore density is negatively correlated with increased stiffness of the Schlemm’s canal endothelium in glaucomatous eyes (11, 12). Thus, it is likely that physical properties of Schlemm’s canal cells themselves, and/or their underlying basal lamina, play a central role in modulating resistance and IOP homeostasis (13). However, how Schlemm’s canal endothelial stiffness, pore number, and outflow resistance are regulated at the molecular level, and how they might become dysregulated to increase resistance in primary open-angle glaucoma, remain topics of ongoing research.

Schlemm’s canal is a unique vessel with characteristics of lymphatics, such as a discontinuous basement membrane and basal-to-apical transendothelial flow, and those of a venule, including a continuous network of cell-cell junctions (14, 15). Like other hybrid vessels (16–20), Schlemm’s canal is derived from blood vascular endothelium and acquires a lymphatic-like phenotype during development (21). However, how lymphatic characteristics impact function remains unclear. Lymphatic capillaries are highly permeable as befits their function (22). While Schlemm’s canal endothelial cells lack the unique junctional morphology of lymphatic capillaries (21, 23), the canal inner wall has one of the highest hydraulic conductivities of any endothelium in the human body (24), suggesting that lymphatic gene expression may facilitate increased permeability.

The lymphatic-like “hybrid” vascular phenotype of Schlemm’s canal cells is defined at the molecular level by the lymphatic master transcription factor PROX1 (21, 25–27). Developmental deletion or blockade of PROX1 or the typically lymphatic receptor tyrosine kinase FLT4 (also known as VEGFR3) results in an attenuated canal with disorganized morphology (21, 25, 28, 29). However, it is unknown whether PROX1 regulates the same transcriptional targets in the canal as in lymphatic vessels, or whether it has a continuing role in the adult eye. Other developmental pathways, including VEGFA/VEGFR2 and ANGPT1/TEK, regulate IOP homeostasis throughout life (21, 30–33), and lymphatic gene expression is maintained in the mature Schlemm’s canal, supporting continued importance (21, 25, 29). As the elevated pressure characteristic of glaucoma is associated with a loss of tissue permeability, we hypothesized that this might be related to the lymphatic character of the Schlemm’s canal endothelium and its regulation by PROX1. Although a previous report did not observe IOP elevation 2 weeks after endothelial *Prox1* deletion in adult mice (28), these animals died within 3 weeks, leaving it unknown whether lymphatic phenotypes were dispensable in the adult canal or whether dysfunction developed over a longer timeline, consistent with human glaucoma (28).

We have previously developed a highly specific nanocarrier platform composed of the amphiphilic block copolymer poly(ethylene glycol)-*b*-poly(propylene sulfide) (PEG-*b*-PPS) (34) for targeted drug delivery to the Schlemm’s canal endothelium (35, 36). Here, we adapted the platform to deliver 4OH-tamoxifen (4OHT), a tamoxifen metabolite that is commonly used to induce recombination in CreERT2-expressing mouse strains (37). By delivering 4OHT nanocarriers to a single eye, this system allowed us to induce deletion within an individual Schlemm’s canal and obtain same-animal controls by injecting empty nanocarriers in contralateral eyes.

After validation, targeted 4OHT-loaded nanocarriers were used to ablate Schlemm’s canal lymphatic phenotypes via deletion of *Prox1* from adult Schlemm’s canal, bypassing lethal systemic phenotypes. Within 4 weeks, we observed specific, reproducible IOP elevation that persisted throughout life, thus identifying a critical role for PROX1 and Schlemm’s canal’s lymphatic phenotype in IOP homeostasis while also generating an adult-onset model of ocular hypertension.

## Results

### Schlemm’s canal endothelial cells have a hybrid lymphatic-like phenotype that is mediated in mice and humans by PROX1.

**Figure 1:**
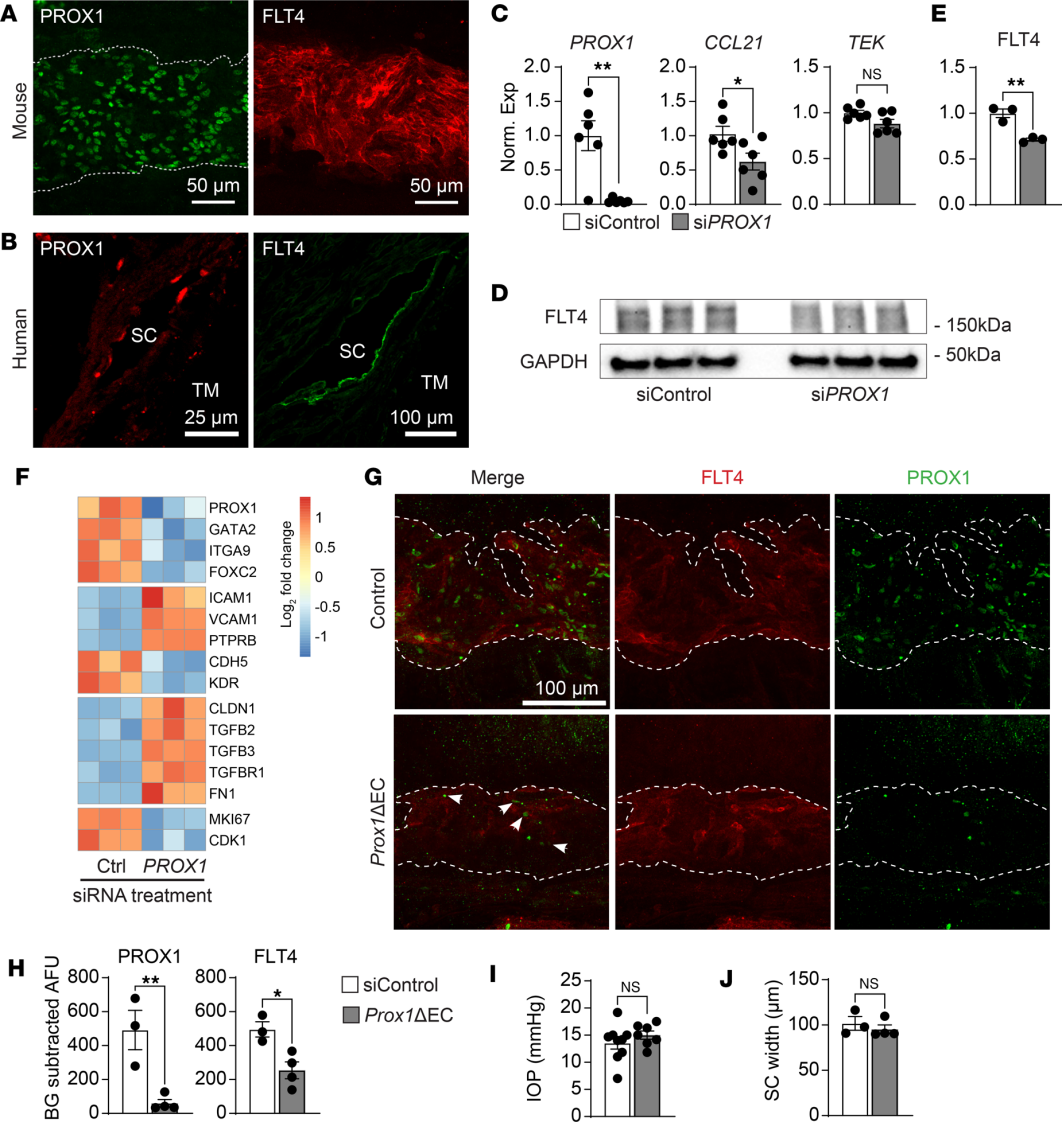
Human and mouse Schlemm’s canal endothelial cells share a lymphatic-like hybrid phenotype mediated by PROX1. (**A** and **B**) Whole-mount immunostaining of mouse (**A**) and cryosections of human (**B**) Schlemm’s canals revealed robust expression of the lymphatic markers PROX1 and FLT4. Scale bars: 50 μm (**A**), 25 μm (**B**, left), 100 μm (**B**, right). (**C**) *PROX1* and *CCL21* expression was reduced in primary human Schlemm’s canal endothelial cells treated with PROX1 siRNA, while levels of the universally expressed endothelial gene *TEK* were unchanged when measured by real-time reverse transcriptase PCR (siControl, *n* = 6; si*PROX1*, *n* = 6). (**D**, quantified in **E**) Western blot revealed reduced expression of FLT4 protein in si*PROX1*-treated human Schlemm’s canal cells (*n* = 3 per group). (**F**) Reduced expression of lymphatic genes and increased expression of blood endothelial genes were detected in si*PROX1*-treated Schlemm’s canal cells by RNA sequencing, accompanied by increased expression of TGFB-signaling genes and reduction in cell proliferation markers (*n* = 3 per group). (**G**, quantified in **H**) Confocal microscopy of Schlemm’s canal flat mounts revealed reduced PROX1 and FLT4 expression in *Prox1^fl/fl^* *Cdh5-*CreERT2 mice 4 weeks after tamoxifen induction at 8 weeks of age (*Prox1*ΔEC). While *Prox1* deletion was generally robust, some mosaicism was observed, and a small number of PROX1-positive nuclei were observed in *Prox1*ΔEC Schlemm’s canal (white arrowheads; *Prox1*ΔEC, *n* = 3; Control, *n* = 4). Dashed lines in **G** outline Schlemm’s canal. BG, background; AFU, arbitrary fluorescence units. Scale bar: 100 μm. (**I** and **J**) No significant change in IOP (*Prox1*ΔEC, *n* = 9; Control, *n* = 7) (**I**) or Schlemm’s canal (SC) width (*Prox1*ΔEC, *n* = 3; Control, *n* = 4) (**J**) was measured 4 weeks after whole-body tamoxifen induction. *P < 0.05, **P < 0.01 as determined by 2-tailed, unpaired Student’s *t* test. Error bars in **C**, **E**, **H**, and **I** indicate ± SEM, while each point denotes an independent biological replicate.

The hybrid identity of Schlemm’s canal endothelial cells has been reported in the mouse eye at both mRNA and protein levels (21, 25, 27, 29, 38, 39). In the human eye, while lymphatic gene expression has been identified by single-cell RNA sequencing (38, 40, 41), it has been less well characterized at the protein level, and conflicting reports have been published (25, 42). To verify the lymphatic identity of this endothelium and determine whether it is regulated by PROX1, we performed immunostaining of mouse and human canals. Confocal microscopy of mouse eye whole mounts (Figure 1A) and cryosections from human corneal rim tissue obtained after transplant surgery (Figure 1B) confirmed that the canal of both species expressed lymphatic markers PROX1 and FLT4, but not LYVE1 (Supplemental Figure 1; supplemental material available online with this article; https://doi.org/10.1172/jci.insight.203711DS1), confirming their hybrid identity. PROX1 expression was negatively correlated with donor age (Supplemental Figure 2A), consistent with reported findings in the mouse eye (28).

As PROX1 directly regulates the fate of lymphatic endothelial cells, we speculated that it plays a similar role in Schlemm’s canal. To test this directly, we treated primary human Schlemm’s canal cells with a previously validated siRNA targeting *PROX1* (43). Seventy-two hours after treatment, real-time reverse transcription PCR was used to confirm *PROX1* knockdown (Figure 1C). Expression of *CCL21* and *FLT4*, additional lymphatic markers expressed by Schlemm’s canal endothelial cells (Figure 1, C–E) (25), was also suppressed, suggesting that loss of PROX1 resulted in a general reduction of lymphatic gene expression. Expression of *TEK*, an endothelial receptor tyrosine kinase expressed in both blood and lymphatic endothelial cells, was unchanged (Figure 1C). RNA sequencing revealed downregulation of additional canal-expressed lymphatic genes in si*PROX1*-treated cells, including *ITGA9* and *GATA2* (Figure 1F and Supplemental Dataset 1). In contrast, a subset of blood-endothelial-specific genes, including *ICAM1*, *VCAM1*, and *PTPRB*, were upregulated. Together these findings suggested that, as in lymphatic endothelial cells (44), the lymphatic transcriptional program in Schlemm’s canal endothelial cells was mediated by PROX1.

To understand the role of PROX1 in mediating the Schlemm’s canal hybrid phenotype in vivo, we next turned to an endothelial cell–specific *Prox1* knockout mouse model (45). *Prox1^fl/fl^* *Cdh5-*CreERT2 mice were generated and induced with tamoxifen at 8 weeks of age to obtain animals lacking *Prox1* in all endothelial cells, including those in Schlemm’s canal (*Prox1*ΔEC). Four weeks after induction, immunostaining revealed robust PROX1 ablation, although some mosaicism was observed and occasional endothelial cells retained PROX1 (Figure 1G, quantified in Figure 1H). FLT4 staining was also reduced, consistent with our in vitro data and the well-known role of PROX1 in regulating FLT4 expression in lymphatic endothelial cells (44). IOP measurement by rebound tonometry (Figure 1I) revealed normal IOP, and no change in Schlemm’s canal size was observed when measured using immunofluorescence confocal microscopy in canal whole mounts (Figure 1J).

Lack of IOP elevation 2 weeks after whole-endothelial *Prox1* deletion has been previously reported (28), and, as in those reports, we were unable to track the IOP of *Prox1*ΔEC mice beyond 4 weeks because of intestinal hemorrhage and lethality, likely caused by the role of lymphatic vessels in maintaining the gut epithelium (28, 46). While these data confirmed that PROX1 regulated Schlemm’s canal’s lymphatic gene expression, we remained skeptical that the hybrid identity was unnecessary for IOP homeostasis — especially as the decrease in PROX1 expression with age in mice (28) and humans (Supplemental Figure 2A) is consistent with an age-related increase in outflow resistance that occurs in humans (47). Thus, we speculated that the short survival time of *Prox1*ΔEC mice might be insufficient to observe an IOP phenotype, or that whole-endothelial *Prox1* deletion caused confounding phenotypes that lowered IOP or prevented accurate tonometric measurements.

To bypass systemic phenotypes, we determined that Schlemm’s canal–specific deletion would be required. However, no canal-specific Cre-expressing mouse line has been described, necessitating the development of a new approach. We therefore employed the PEG-*b*-PPS nanocarrier delivery system previously developed by our group in order to selectively deliver the active metabolite 4OHT directly to Schlemm’s canal endothelial cells of *Prox1^fl/fl^* *Cdh5-*CreERT2 mice to induce Schlemm’s canal–specific Cre-mediated recombination.

### Targeted 4OHT nanocarriers specifically induce gene deletion in Schlemm’s canal endothelial cells.

**Figure 2:**
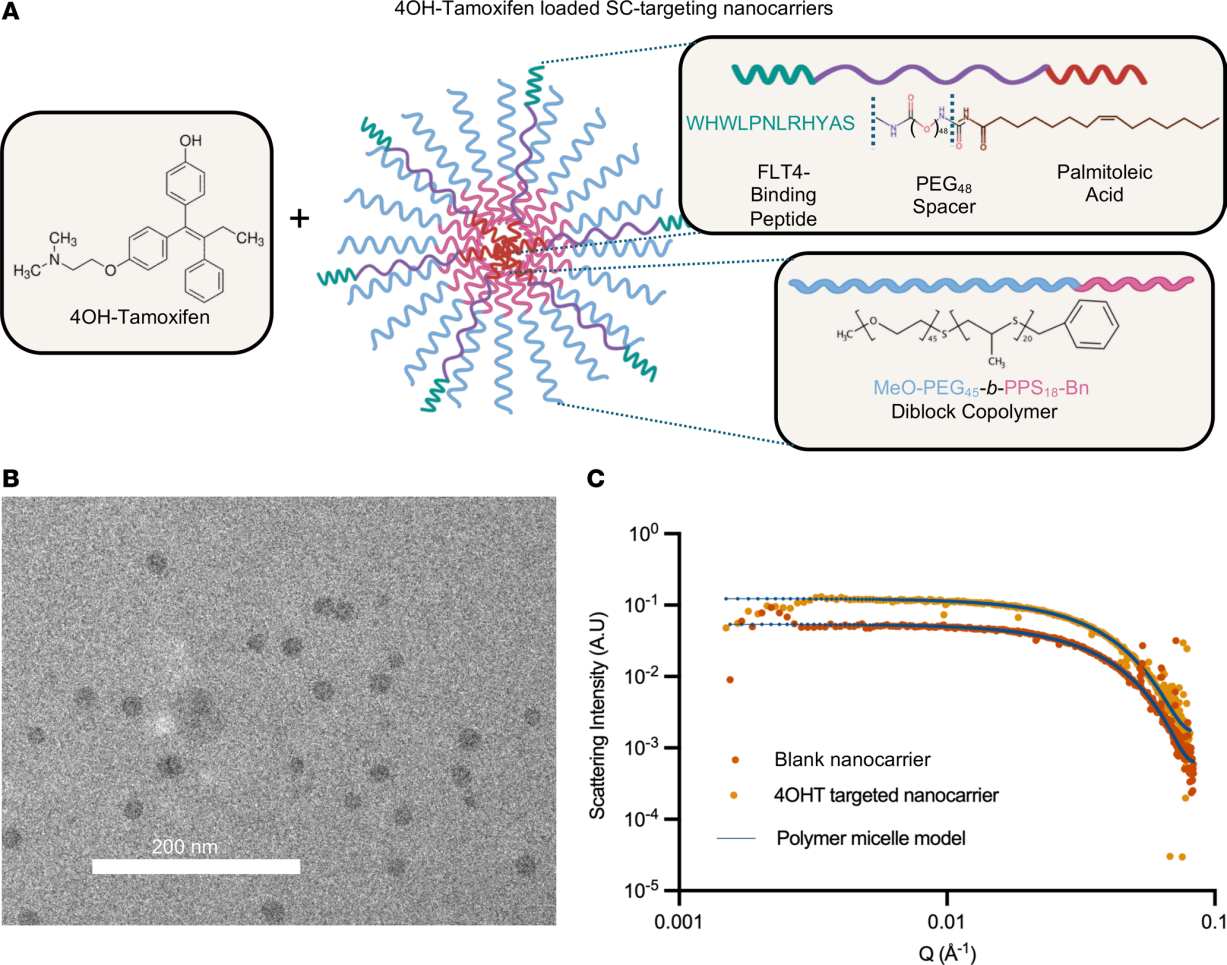
Characterization of targeted and untargeted 4OH-tamoxifen–loaded nanocarriers. (**A**) Schematic illustrating how PEG-*b*-PPS targeted nanocarriers are generated and loaded with 4OH-tamoxifen (4OHT). (**B** and **C**) Micellar morphology for 4OHT nanocarriers was characterized by cryogenic transmission electron microscopy (**B**) and small-angle x-ray scattering (*n* = 2) (**C**). The targeted PEG-*b*-PPS nanocarrier platform was previously validated and optimized for selective drug delivery to Schlemm’s canal cells in vitro and in vivo (36). Scale bar: 200 nm.

Modifying a PEG-*b*-PPS delivery system that was previously developed by our group (34–36), we designed a targeted nanocarrier system to selectively deliver 4OHT to Schlemm’s canal endothelial cells in vivo to achieve Schlemm’s canal–specific, Cre-mediated gene deletion. Combined with endothelial cell–specific expression of Cre-ERT2 in *Cdh5-*CreERT2 mice, we predicted that this system would allow robust, 2-level selectivity for the Schlemm’s canal endothelium. Micellar nanocarriers were generated, loaded with 4OHT, and decorated with VEGFC-derived peptide-lipid targeting constructs (PG48), which we have previously optimized for FLT4 binding and selective delivery to the Schlemm’s canal endothelium (Figure 2A) (35, 36). Unlike unmetabolized tamoxifen that must circulate for processing by cytochrome P450 in the liver (48), 4OHT is active immediately in cells where it is delivered, making it the optimal cargo for this application. 4OHT nanocarriers exhibited a stable monodisperse particle size distribution (polydispersity index ≤ 0.2) with a diameter of 17–22 nm when assessed by cryogenic electron microscopy and dynamic light scattering (Figure 2B, Supplemental Figure 3A, and Table 1). ζ-Potential was determined by electrophoretic light scattering, indicating a neutral surface charge (Table 1). Spherical morphology was confirmed by cryogenic transmission electron microscopy (Figure 2B) and small-angle x-ray scattering (Figure 2C) and was unaffected by addition of 4OHT and targeting peptides. Encapsulation efficiency of 4OHT was 47%–54% (Table 1) with a resulting concentration of 80–120 μg/mL encapsulated 4OHT in injectable formulations. No cytotoxicity was observed in cultured HUVECs treated with nanocarrier concentrations up to 2 μM 4OHT (Supplemental Figure 3B). Validating the targeting approach, flow cytometry demonstrated that VEGFC-derived targeting peptides increased uptake of DiI-labeled nanocarriers by primary human Schlemm’s canal cells but not HUVECs, which do not express FLT4 (Supplemental Figure 3C).

### 4OHT nanocarriers specifically induce gene deletion in Schlemm’s canal in vivo.

**Figure 3:**
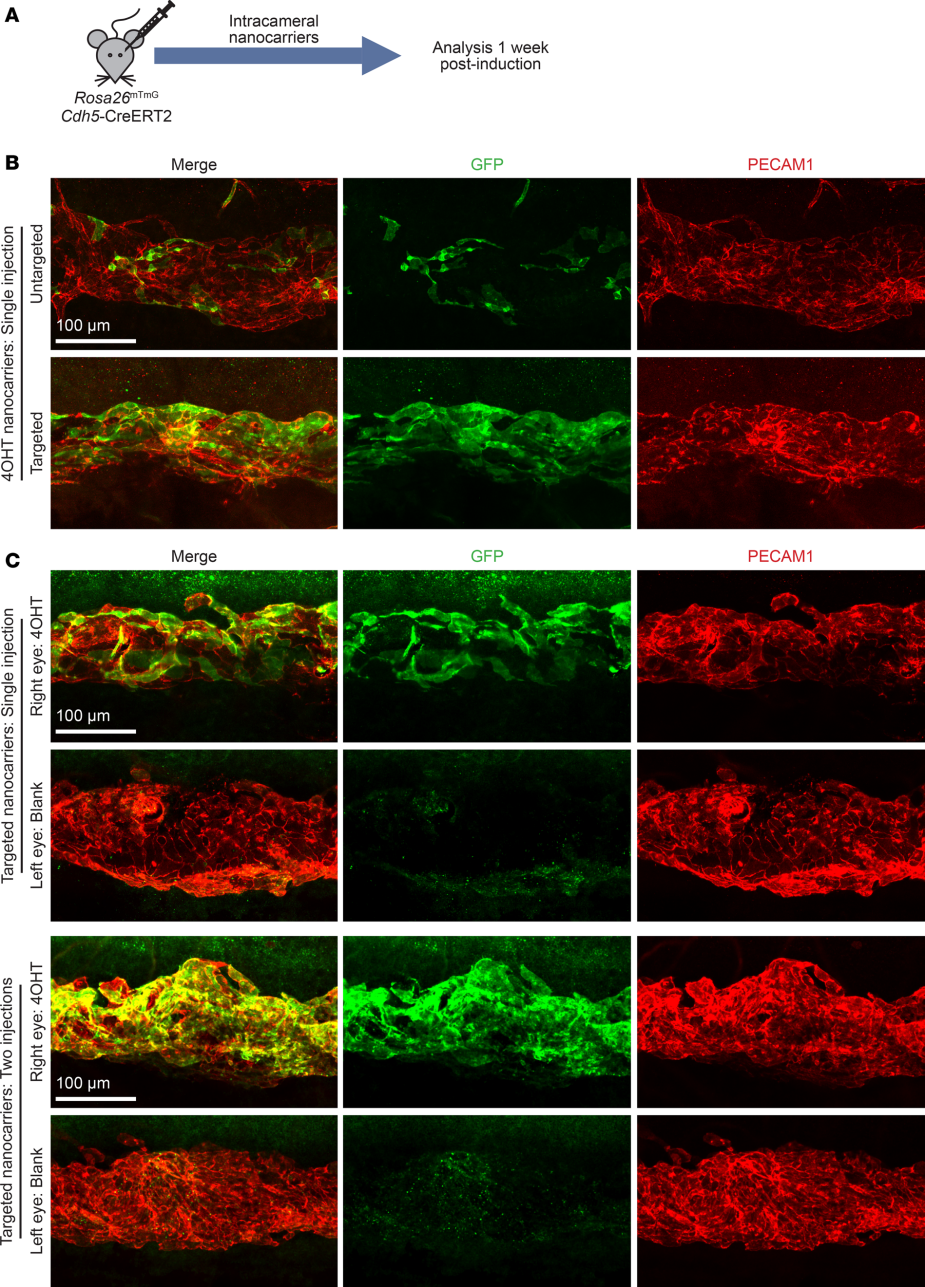
4OHT-loaded, Schlemm’s canal–targeted nanocarriers induce robust Cre-mediated recombination. (**A**) Schematic of experimental timeline used for nanocarrier induction. (**B**) Seven days after intracameral infusion of untargeted 4OHT-loaded nanocarriers, only sporadic recombination of Schlemm’s canal endothelial cells was observed in *Rosa26^mTmG^* *Cdh5*-CreERT2 mice (GFP expression). In contrast, robust recombination was induced by nanocarriers labeled with FLT4-targeting peptide, indicating enhanced uptake by the Schlemm’s canal endothelium. Scale bar: 100 μm. (**C**) Schlemm’s canal–specific recombination efficiency was further increased by a second injection of FLT4-targeting nanocarriers 24 hours after the first. No recombination was observed in Schlemm’s canal of contralateral eyes that received infusions of identical targeted nanocarriers lacking 4OHT cargo (blank nanocarriers) as a negative control, confirming specificity of the system and lack of systemic recombination following nanocarrier treatment. Scale bars: 100 μm.

To determine whether 4OHT nanocarriers could induce high efficiency, Schlemm’s canal–specific recombination and whether FLT4 targeting was as effective in vivo as in our in vitro system, we administered parallel intracameral injections of targeted and untargeted 4OHT nanocarriers to eyes of *Rosa26^mTmG^* *Cdh5*-CreERT2 reporter mice (Figure 3A). Eyes were collected for analysis 7 days later. Although sporadic 4OHT-induced Cre-mediated recombination was observed in eyes receiving untargeted, 4OHT-loaded nanocarriers, the majority of the endothelial cells remained unrecombined, indicating low-efficiency uptake (Figure 3B). In contrast, recombination efficiency was markedly increased in eyes treated with FLT4-targeted 4OHT nanocarriers, confirming effectiveness of the targeting strategy (Figure 3B).

We next tested repeat nanocarrier injections as a tool to increase recombination efficiency (Figure 3C). Following a second injection 24 hours after the first, we observed nearly complete labeling of the Schlemm’s canal endothelium. Despite robust recombination within the eye receiving 4OHT nanocarriers, no recombination was observed in Schlemm’s canal of contralateral eyes that received identical FLT4-targeted empty (blank) nanocarriers as a control (Figure 3C), or within retinal or choroidal capillaries of either eye (Supplemental Figure 4), confirming specificity of the targeting approach and lack of systemic recombination. Mosaic recombination was seen in distal outflow vessels of eyes receiving 4OHT-loaded nanocarriers, suggesting that some nanocarriers flowed through the canal without uptake by Schlemm’s canal endothelium; and in limbal lymphatic capillaries, consistent with their known expression of FLT4 (Supplemental Figure 5, A and B). Outside the eye, while we observed GFP expression in some endothelial cells of the liver, kidney, and lung (Supplemental Figure 6), recombination levels were similar to those in untreated animals, indicating that recombination in these tissues was due to leakiness of Cre activity and not off-target nanocarrier delivery.

### Schlemm’s canal–specific Prox1 deletion causes ocular hypertension in adult mice.

**Figure 4:**
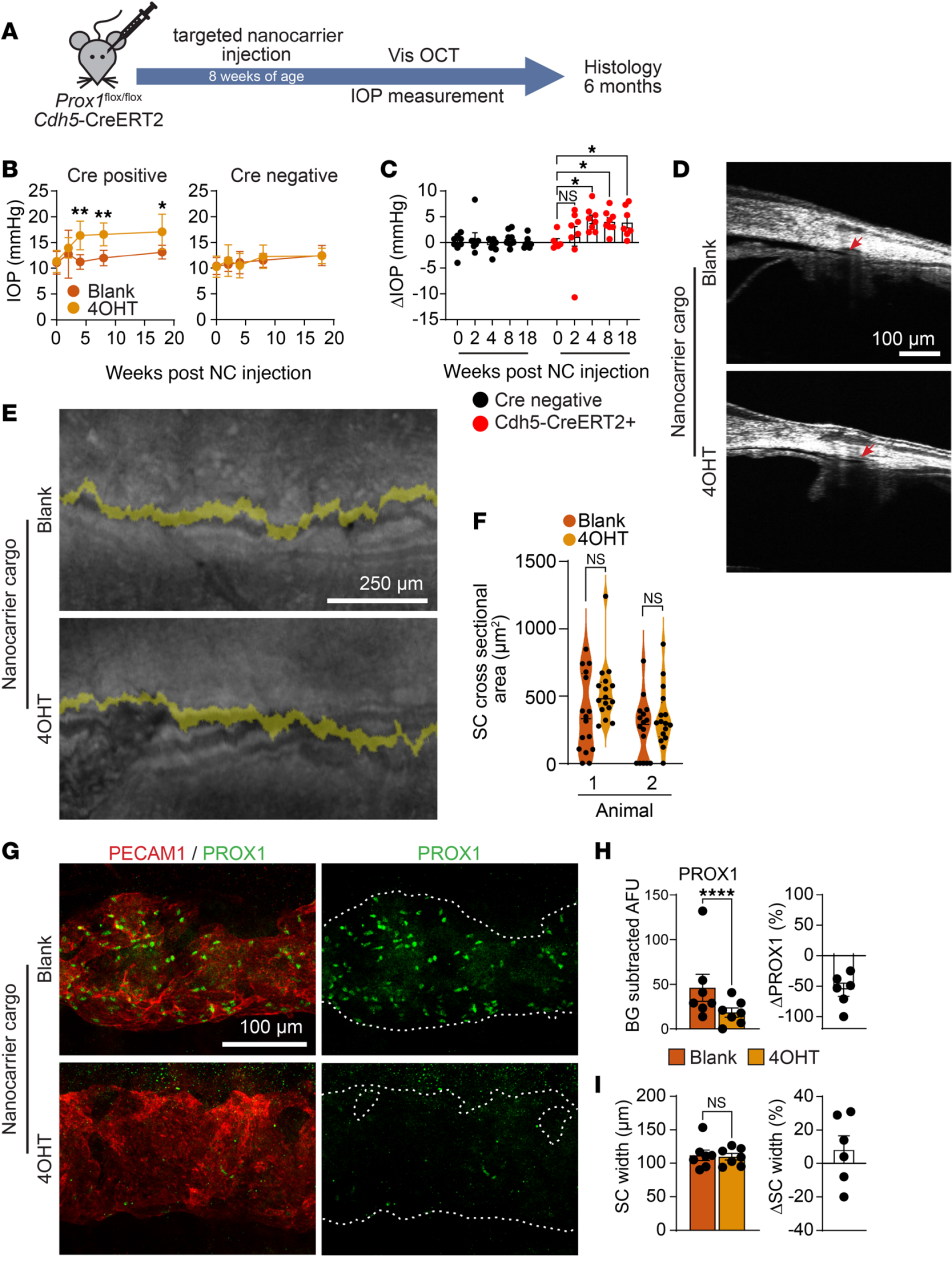
Schlemm’s canal–specific *Prox1* deletion leads to ocular hypertension. (**A**) Schematic of experimental timeline used for targeted nanocarrier induction of *Prox1* deletion. (**B** and **C**) Four weeks after targeted nanocarrier induction, IOP elevation was observed in eyes of *Prox1^fl/fl^* *Cdh5*-CreERT2 mice receiving targeted 4OHT nanocarriers in comparison with contralateral eyes receiving identical targeted empty (blank) nanocarriers. IOP elevation persisted throughout the duration of the experiment. No IOP elevation was seen in identically treated Cre-negative mice (Cre^+^, *n* = 8; Cre^–^, *n* = 8). NC, nanocarrier. (**D** and **E**) In vivo visible-light OCT imaging 12 weeks after nanocarrier-mediated *Prox1* deletion revealed no change in canal size on B-scans (**D**) or longitudinal reconstructions (pseudo-colored in yellow) of Schlemm’s canal (**E**). Scale bars: 100 μm (**D**), 250 μm (**E**). (**F**) Comparison of luminal area by 16 individual OCT B-scans captured around the circumference of Schlemm’s canal showed no difference in canal size between matched 4OHT- and blank nanocarrier–treated eyes of *Cdh5*-CreERT2–positive mice. (**G**) PROX1 expression and canal morphology were examined in Schlemm’s canal flat mounts collected 6 months after nanocarrier induction by confocal microscopy. Dashed lines in PROX1 panels indicate the outline of PECAM1-positive Schlemm’s canal. Scale bar: 100 μm. (**H**) Compared with contralateral control eyes, PROX1 expression was significantly reduced in eyes treated with 4OHT nanocarriers. BG, background; AFU, arbitrary fluorescence units. (**I**) No significant change in Schlemm’s canal (SC) size was observed in 4OHT nanocarrier–treated eyes (*n* = 6). *P < 0.05, **P < 0.01, ****P < 0.0001 as determined by 2-way ANOVA followed by Bonferroni’s post-tests (**B**, **C** and **F**) or 2-tailed paired Student’s *t* test (**H** and **I**). Error bars in **B**, **C**, **H**, and **I** indicate ± SEM, while each point represents an independent biological replicate. Each point in **F** represents a single measurement captured along the length of Schlemm’s canal, while each violin represents a single eye.

Following validation of targeted 4OHT nanocarriers, we examined the long-term role of PROX1 in maintaining IOP homeostasis. We generated cohorts of *Prox1^fl/fl^* *Cdh5*-CreERT2 mice with Cre-negative littermate controls. At 8 weeks of age, Schlemm’s canal–specific *Prox1* deletion was induced in randomized eyes with Schlemm’s canal–targeted 4OHT nanocarriers. Contralateral eyes received identical injections of targeted empty (blank) nanocarriers as a control (Figure 4A). At baseline, we observed no difference in IOP between eyes (ΔIOP_baseline_ = 0.2 ± 0.6 mmHg, *P >* 0.9), but beginning 4 weeks after induction, IOP in 4OHT-treated eyes was significantly elevated in comparison with same-animal control eyes (ΔIOP_4wks_ = 4.5 ± 0.8 mmHg, *P* < 0.005) (Figure 4, B and C). No IOP difference was observed between eyes in Cre-negative littermates insensitive to tamoxifen (ΔIOP_4wks_ = –0.6 ± 0.5 mmHg, *P >* 0.9). IOP remained elevated for 18 weeks, as long as the animals were maintained. In an independent replication cohort, elevated IOP was observed for 24 weeks, again as long as the animals were maintained (ΔIOP_24wks_ = 4.4 ± 1.3 mmHg, *P* < 0.001; Supplemental Figure 7). Surprisingly, given increased IOP and the central role of PROX1 in Schlemm’s canal development, in vivo visible-light optical coherence tomography imaging performed 12 weeks after induction revealed no difference in Schlemm’s canal size or morphology between *Prox1*-knockout and same-animal contralateral control eyes (Figure 4, D and E, quantified in Figure 4F).

**Figure 5:**
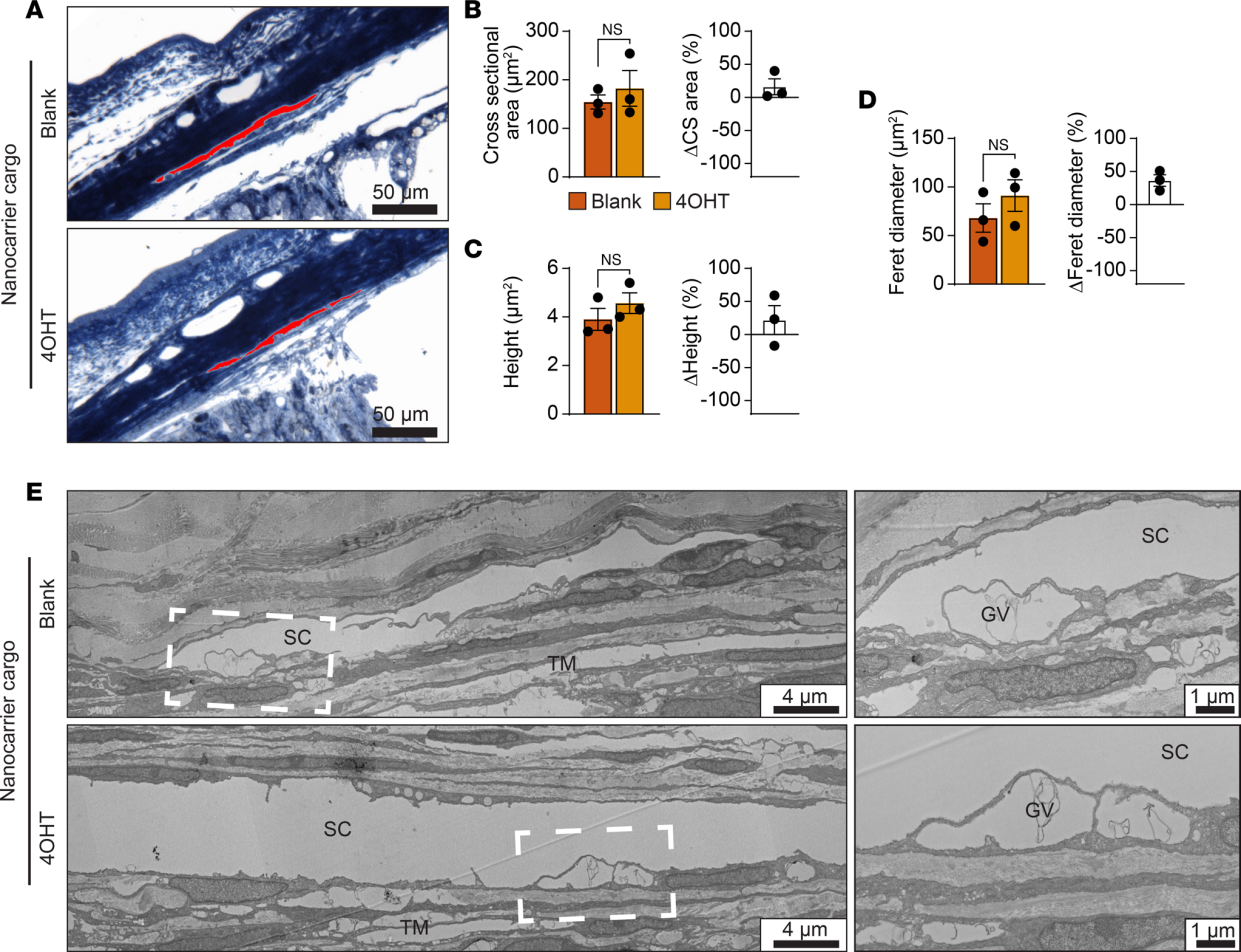
Light and electron microscopy reveals normal Schlemm’s canal morphology in *Prox1*-knockout Schlemm’s canal. (**A**–**D**) Representative toluidine blue–stained semithin cross sections of Schlemm’s canal from *Prox1^fl/fl^* *Cdh5*-CreERT2 eyes 12 weeks after treatment with blank or 4OHT nanocarriers (**A**). Scale bars: 50 μm. Semithin sections were imaged by light microscopy and used to measure Schlemm’s canal cross-sectional area (**B**), canal height (depth) (**C**), and Feret diameter (longest axis; *n* = 3 pairs of eyes from *Cdh5*-CreERT2–positive nanocarrier-treated animals) (**D**). Schlemm’s canal lumen is indicated by red shading. In comparison with same-animal contralateral control eyes, no significant difference in canal size or morphology was observed. (**E**) Representative transmission electron microscopy images from the inferior quadrant of matching eyes treated with 4OHT or blank nanocarriers from a *Prox1^fl/fl^* *Cdh5*-CreERT2 mouse revealed no differences in Schlemm’s canal (SC) inner wall or juxtacanalicular meshwork morphology 12 weeks after nanocarrier injection. Giant vacuoles (GV) were observed in the inner wall of both 4OHT-treated and contralateral control eyes. TM, trabecular meshwork. Scale bars: 4 μm (left), 1 μm (right). Statistical comparisons in **B**–**D** were performed using a Bonferroni-corrected 2-tailed, paired Student’s *t* test. Error bars in **B**–**D** indicate ± SEM, while each point represents an independent biological replicate.

Following longitudinal IOP measurements, enucleated eyes were stained and prepared for immunofluorescent imaging (whole mounts) and for light and electron microscopy (cross sections). Compared with contralateral controls, confocal microscopy confirmed reduced PROX1 immunostaining in canals of targeted 4OHT nanocarrier–treated eyes (56% ± 10.8% reduction, *P* < 0.001; Figure 4G, quantified in Figure 4H). Schlemm’s canal size, as measured by PECAM1-positive immunofluorescence, was unchanged (Figure 4I). At 12 weeks of age, light microscopy in a separate group of animals revealed no differences in gross canal morphology or geometric parameters between *Prox1*-knockout and contralateral control eyes (Figure 5, A–D). Similarly, when imaged using transmission electron microscopy (Figure 5E), we did not observe differences in the morphology of Schlemm’s canal inner wall or juxtacanalicular connective tissue. Giant vacuoles were observed in the Schlemm’s canal of both *Prox1*-knockout and control eyes.

These findings suggested that increased IOP seen following *Prox1* deletion was due to alterations in outflow function of the targeted Schlemm’s canal cells and/or in their communication and interactions with neighboring cells or extracellular matrix, rather than canal degeneration or gross morphological changes. Furthermore, they confirmed the efficacy of FLT4-targeted 4OHT nanocarriers for gene deletion within Schlemm’s canal and demonstrated that PROX1-mediated lymphatic hybrid phenotypes were essential for IOP homeostasis.

In contrast to the long-term persistence of Schlemm’s canal following *Prox1* deletion, we observed a reduced number of limbal lymphatic capillaries in 4OHT nanocarrier–treated eyes, which were targeted by our FLT4-based strategy in addition to Schlemm’s canal (Supplemental Figure 8), consistent with their requirement for ongoing PROX1 activity (44, 49). Normal PROX1 expression was seen in remaining lymphatics, suggesting that deletion efficacy was lower in these vessels than in Schlemm’s canal — perhaps because the limbal lymphatics do not connect to the anterior chamber and lacked a direct route for nanocarrier entry.

### Endothelial cell–specific Flt4-knockout mice induced as adults have normal IOP.

**Figure 6:**
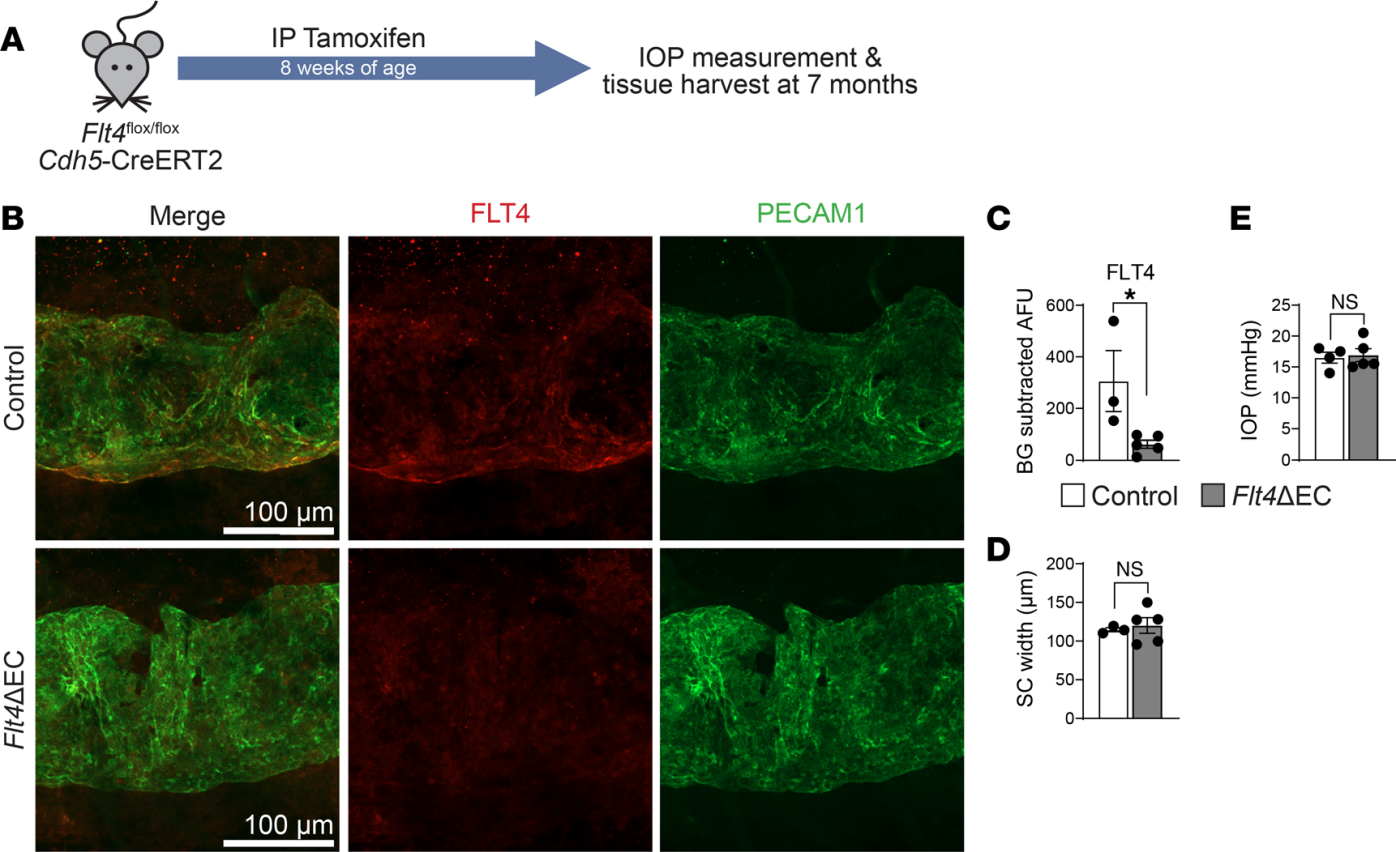
FLT4 is not required for Schlemm’s canal maintenance or IOP homeostasis in adult mice. (**A**) Experimental outline used for generation and analysis of endothelial cell–specific *Flt4*-knockout mice (*Flt4*ΔEC). (**B**, quantified in **C** and **D**) Confocal microscopy revealed loss of FLT4 immunostaining and similar PECAM1-positive Schlemm’s canal size 5 months after *Flt4* deletion (Control, *n* = 5; *Flt4*ΔEC, *n* = 3). BG, background; AFU, arbitrary fluorescence units. Scale bars: 100 μm. (**E**) No difference in IOP was observed between *Flt4*ΔEC mice and control littermates at 7 months of age (Control, *n* = 5; *Flt4*ΔEC, *n* = 4). Statistical comparisons in **C**–**E** were performed using a 2-tailed Student’s *t* test. *P < 0.05. Error bars indicate ± SEM, while each point represents an independent biological replicate.

While PROX1 has many direct transcriptional targets, one of the best characterized is *Flt4*. *PROX1* knockdown in cultured lymphatic endothelial cells (44) and in primary human Schlemm’s canal cells (Figure 1) led to reduction of *FLT4*, and *PROX1* overexpression drives ectopic *FLT4* expression in blood vascular endothelial cells (44). In mouse lymphatic vessels, *Prox1* deletion leads to loss of FLT4 and the lymphatic phenotype overall (50, 51). Accordingly, as FLT4 is essential for Schlemm’s canal development and its ligand VEGFC induces angiogenic sprouting from the adult canal (25), we speculated that FLT4 downregulation may be the cause of IOP elevation in *Prox1*-knockout eyes. Therefore, we generated mice with endothelial cell–specific *Flt4* knockout using *Cdh5*-CreERT2 (*Flt4*ΔEC), and induced deletion by tamoxifen injection at 8 weeks of age (Figure 6A).

Twenty weeks after tamoxifen administration, confocal microscopy revealed loss of FLT4 immunostaining from Schlemm’s canal in *Flt4*ΔEC mice (Figure 6B, quantified in Figure 6C) with no change in PECAM1-positive Schlemm’s canal size or morphology (Figure 6D). No difference in IOP was observed between *Flt4*ΔEC mice and Cre-negative control littermates (ΔIOP = 0.4 mmHg, *P >* 0.7; Figure 6E). These data indicated that, while FLT4 is essential for Schlemm’s canal development (25), it was dispensable in the adult canal and FLT4 downregulation was not responsible for the IOP elevation seen in *Prox1*ΔSC eyes.

## Discussion

It has long been known (6, 7) that the ocular hypertension characteristic of primary open-angle glaucoma is due to increased resistance to the flow of aqueous humor through the outflow pathway in the eye. While the cause of this increased resistance remains incompletely understood, several factors implicate the inner wall region of Schlemm’s canal of glaucomatous eyes, including decreased pore density (9, 10), increased cellular (52) and tissue stiffness (12, 53), and increased pressure drop in this region (12). As there is also evidence that Schlemm’s canal endothelium modulates outflow resistance and thereby IOP in the normal eye (8), here, we focused on how this modulation might occur.

PROX1 is crucial for Schlemm’s canal development, but its systemic importance within the lymphatic endothelium has prevented long-term studies of its role in the adult eye and masked any ongoing role in IOP regulation (28). Here, by deleting *Prox1* selectively from Schlemm’s canal endothelium, our targeted nanocarrier approach allowed us to study knockout eyes over a longer period and revealed an unappreciated but essential role for PROX1 in IOP homeostasis.

Schlemm’s canal originates through sprouting angiogenesis from blood-filled capillaries in the limbal and iridocorneal angle regions of the eye (21, 25). PROX1 expression begins concurrently with canal lumenization, and in turn initiates expression of FLT4 and other lymphatic markers. The molecular identity of true lymphatic vessels is maintained by a signaling loop of PROX1 and FLT4 (44, 49). Without continuous PROX1 transcriptional activity, lymphatic endothelial cells revert toward a venous phenotype and degenerate (49), consistent with the loss of limbal lymphatic capillaries that we observed in *Prox1*-knockout eyes. Conversely, while deletion of *Prox1* from Schlemm’s canal markedly increased IOP, we did not observe canal degeneration or reduction in canal size over timelines up to 6 months.

Together, these findings suggested that the PROX1-mediated hybrid phenotype was essential for IOP homeostasis, but that without PROX1, Schlemm’s canal could indefinitely revert to a less functional vein-like phenotype. This mirrors other PROX1-expressing hybrid vessels (20) and suggests that reprogramming the Schlemm’s canal endothelium to enhance or restore its functional hybrid phenotype may be an effective long-term or permanent strategy for enhancing or restoring outflow. We further showed relevance of this model to the human eye by confirming the presence of PROX1 in human Schlemm’s canal, as predicted by single-cell RNA sequencing (38, 41) and in agreement with the findings of Aspelund et al. (25) but contrary to the report of Birke et al. (42).

While these findings established a clear connection between the hybrid phenotype and function, the mechanistic link is still unclear. Lymphatic capillaries are less stiff (54) and more permeable (22) than veins, characteristics central to the function of Schlemm’s canal. This suggests that elevated IOP in this model may be due to lowered permeability of the inner wall endothelial cells following *Prox1* deletion. How these characteristics are regulated by PROX1 is unknown, but our in vitro studies provided clues. Following siRNA-mediated *PROX1* knockdown, expression of *PTPRB*, the gene encoding the TEK- and VE-cadherin–regulating phosphatase VE-PTP, was increased. *Ptprb* deletion in mice or pharmacological VE-PTP inhibition in mice, rabbits, and humans lowers IOP (55–57), while rare *PTPRB* variants are associated with reduced glaucoma risk (58). *Ptprb*/VE-PTP is highly expressed in blood vascular endothelial cells but is absent in lymphatics (59), consistent with transcriptional suppression by PROX1.

### Schlemm’s canal–specific Prox1-knockout mice as a model of ocular hypertension.

Rodent genetic models of ocular hypertension have provided invaluable insights into glaucoma pathogenesis, genetics, and treatment. However, many existing models are developmental in nature (60), exhibit very high IOP (60), or are associated with ocular inflammation (61) or anterior chamber abnormalities (62) that complicate interpretation. Other models, such as *MYOC^Y437H^* transgenic mice, have been reported to lose pressure elevation over time or exhibit environment-specific effects, limiting their utility (63–65). Here, we report that Schlemm’s canal–specific *Prox1*-knockout mice developed significant IOP elevation in comparison with contralateral control eyes injected with control nanocarriers, and elevated IOP was maintained for at least 6 months. In humans, ocular hypertension is defined as an IOP exceeding 21 mmHg (66). As the population-average IOP in humans is approximately 15 mmHg (67), this corresponds to an average pressure increase of more than 6 mmHg. In our study, baseline pressure was 10.7 ± 0.4 mmHg (Figure 4B) and, 8 weeks after induction, had increased to 16.6 ± 0.8 mmHg in *Prox1*-knockout eyes, a difference of 5.9 mmHg, slightly less than the pressure increase defined as ocular hypertension in humans. Consistent with human ocular hypertension, no structural abnormalities of the mouse aqueous outflow structures were observed. These findings suggest that our model may be valuable for future studies examining the impact of elevated IOP on retinal function or testing IOP-lowering drugs.

### OHT nanocarriers are a valuable tool for targeted gene deletion.

Combining the well-validated endothelial *Cdh5*-CreERT2 mouse strain with tissue-specific 4OHT delivery via a targeted non-inflammatory nanocarrier (36, 68–70) allowed robust gene deletion and increased tissue specificity without ocular toxicity. While adeno-associated viruses (AAVs) have been used in similar applications to generate models of eye disease (33) and for gene therapy (71), ocular inflammation has been observed in patients (72–74) and rodent models (75) following AAV administration. In contrast, the PEG-*b*-PPS platform is non-inflammatory (76), can be customized for cell-specific targeting (36, 68–70), and can be adapted to deliver a wide array of cargoes and combinations thereof (36, 77, 78).

In addition to small diameter (~22 nm) that facilitated passage through the trabecular meshwork, PEG-*b*-PPS nanocarriers are highly efficient at intracellular delivery (68, 79), improving therapeutic effects at lower payload concentrations. This approach enhanced 4OHT efficacy for driving recombination in nearly all targeted Schlemm’s canal cells, while avoiding systemic effects of pan-endothelial *Prox1* deletion. The 4OHT-loaded PEG-*b*-PPS nanocarriers are a promising tool for achieving customizable and geographically targeted investigation of gene expression in other tissues and models, with observed responses being attributed to specific gene manipulation instead of nonspecific inflammation or toxicity from tamoxifen or the delivery vehicle itself.

### Limitations.

Our study has several limitations that deserve investigation beyond the scope of the present article. Prior reports (80) suggest that the level of IOP elevation seen in Schlemm’s canal–specific *Prox1*-knockout eyes is likely to cause retinal ganglion cell loss and glaucoma. However, this remains untested, and it is possible that low baseline IOP seen in *Prox1^fl/fl^* *Cdh5*-CreERT2 mice prior to *Prox1* deletion will provide some protection from optic neuropathy. In addition, while whole-mount immunostaining and penetrance of IOP elevation in nanocarrier-induced *Prox1*-knockout eyes indicated a consistent level of deletion, our Schlemm’s canal–specific approach combined with the presence of nearby PROX1-expressing lymphatic vessels did not permit more sensitive whole-tissue quantification methods such as Western blot or reverse transcriptase PCR. Accordingly, we cannot exclude the possibility that increased deletion efficacy would further elevate IOP — especially as the physical mechanism by which *PROX1* regulates aqueous humor outflow resistance remains unknown. Our previous work (11, 12) highlighted the importance of stiffness of the Schlemm’s canal inner wall, and our finding of loss of the lymphatic phenotype in our ocular hypertension mouse model is consistent with this model. Future studies examining stiffness and gene expression in the canal endothelium after deletion or overexpression of *PROX1* and related genes will help to elucidate the pathogenic mechanism.

Second, studies with *Flt4*-knockout mice were performed using systemic administration of tamoxifen rather than Schlemm’s canal–targeting nanocarriers, as, unlike *Prox1*-knockout mice, these animals are viable if induced during adulthood. While we did not observe elevated IOP following pan-endothelial *Flt4* deletion, *Flt4* is expressed by capillaries of the ciliary body (81) in addition to Schlemm’s canal, and we cannot exclude the possibility that production of aqueous humor was reduced in these animals. Future studies with canal-specific *Flt4* knockouts and measurement of outflow facility are warranted.

### Conclusion.

Schlemm’s canal–specific *Prox1* deletion in adult mice leads to long-lasting ocular hypertension without canal degeneration or morphological changes. These findings demonstrate the importance of the canal’s lymphatic characteristics in the maintenance of normal aqueous humor outflow resistance and highlight the phenotypic plasticity of Schlemm’s canal as a target for next-generation therapies for ocular hypertension. In addition to these mechanistic insights, our studies also demonstrate the power of Schlemm’s canal–specific 4OH-tamoxifen nanocarriers for inducing robust, canal-specific gene deletion without systemic phenotypes, as well as introduce canal-specific *Prox1*-knockout mice as a new model of primary open-angle glaucoma.

## Methods

### Sex as a biological variable.

Animals of both sexes were included in all experiments at approximately equal ratios but were analyzed together. Sex-specific analyses were not conducted.

### Animal generation and husbandry.

Mice were housed at the Center for Comparative Medicine of Northwestern University. Animals were provided with unlimited access to water and standard rodent diet (7912, Teklad) and maintained on a standard 12-hour lighting cycle at a temperature of 21°C–23°C and relative humidity of 30%–70%. To generate litters of *Rosa26^mTmG^* *Cdh5*-CreERT2 mice for analysis, gt(ROSA)26Sortm4(ACTB-tdTomato,-EGFP)Luo/J (*Rosa26^mTmG^*) mice (strain 007576, The Jackson Laboratory) (82) were crossed with Tg(Cdh5-cre/ERT2)^1Rha^ animals carrying the *Cdh5*-CreERT2 transgene (83). *Prox1^fl/fl^* mice were a gift from Guillermo Oliver (Northwestern University) (43, 45), and *Flt4*-floxed mice were a gift from Susan E. Quaggin (Northwestern University) (84). Throughout the study, animals were maintained on a mixed genetic background, and animals of both sexes were included in all experiments. Mice were genotyped by PCR using previously published primers.

### Primary human Schlemm’s canal cell culture.

Primary human Schlemm’s canal cells were a gift from W. Daniel Stamer (Duke University School of Medicine, Durham, North Carolina, USA). For siRNA experiments, normal Schlemm’s canal cells from clones SC-68 and SC-91 were seeded in 6-well plates before transfection in triplicate groups using a previously validated *PROX1*-specific siRNA (016913-00-0005, Dharmacon) (43) or matching scrambled siRNA control (016913-00-0005, Dharmacon) in combination with RNAiMAX reagent (13778075, Invitrogen, Thermo Fisher Scientific) according to the manufacturer’s instructions. Seventy-two hours after treatment, total RNA was collected using TRIzol reagent (15596026, Thermo Fisher Scientific) and purified (RNeasy MinElute cleanup kit, QIAGEN) before generation of cDNA for real-time PCR or RNA sequencing.

### HUVEC cell culture.

Human umbilical vein endothelial cells (HUVECs) from pooled donors were purchased from Lonza. All primary cells used in these studies were used at passage 4. HUVECs were cultured in Endothelial Cell Growth Basal Medium-2 (EBM-2, Lonza) supplemented with FBS and Endothelial Cell Growth Medium-2 (EGM-2) BulletKit (Lonza) optimized for HUVEC culture. All cells were cultured at 37°C, 5% CO_2_ in T25 flasks.

### Analysis of human corneal rims.

Surplus corneal rim tissue was obtained after corneal transplant surgery, fixed (4% formaldehyde), and cryosectioned using standard techniques. Tissues recovered and preserved for transplant within 15 hours of death were accepted for analysis; tissue with longer recovery times showed no PROX1 expression (data not shown). No restrictions were placed on the duration of storage in cornea culture medium prior to transplant surgery. Cryosections were washed and blocked (5% donkey serum, 2.5% bovine serum albumin [BSA] in TBS containing 0.5% Triton X-100) before overnight incubation with primary antibodies at 4°C. Sections were then washed and incubated with appropriate Alexa Fluor–labeled secondary antibodies. To quantify PROX1 expression within Schlemm’s canal, sections were costained using antibodies against PROX1 and the endothelial transcription factor ERG, and QuPath software (https://doi.org/10.1038/s41598-017-17204-5) was used to quantify PROX1 fluorescence intensity in ERG-positive Schlemm’s canal nuclei and background intensity in non-endothelial ERG-negative, DAPI-positive nuclei in other cells of the iridocorneal angle. Identically prepared sections from human pterygium tissue were used as a positive control for LYVE1 staining. A complete list of primary antibodies used is provided in Supplemental Table 1.

### Real-time quantitative PCR.

After RNA purification, cDNA was prepared using the iScript Kit (Bio-Rad Laboratories) according to the manufacturer’s instructions. Real-time PCR was then performed using a QuantStudio 3 instrument (Thermo Fisher Scientific) and Power SYBR Green Master Mix (Thermo Fisher Scientific). The following primers were used: GAPDH forward: 5′-AAGGTCATCCCAGAGCTGAA-3′; GAPDH reverse: 5′-CTGCTTCACCACCTTCTTGA-3′; PROX1 forward: 5′-GAGCCTCCGTGGAACTCA-3′; PROX1 reverse: 5′-TGGGCACAGCTCAAGAATC-3′; TEK forward: 5′-CCCCTATGGGTGTTCCTGT-3′; TEK reverse: 5′-GCTTACAATCTGGCCCGTAA-3′; CCL21 forward: 5′-CGCAGCTACCGGAAGCAG-3′; CCL21 reverse: 5′-CTGCCTGAGAGCGCTTGC-3′.

### Western blot.

After siRNA transfection as described above, triplicate samples of primary human Schlemm’s canal cells (clone SC-68) were lysed using Laemmli sample buffer containing 100 mM DTT and separated using a 4%–15% acrylamide minigel (TGX, Bio-Rad Laboratories). Proteins were transferred onto a PVDF membrane (Bio-Rad) and blocked (5% BSA in TBS, pH 7.5, with 0.05% Tween 20, 1 hour at room temperature) before incubation overnight with appropriate primary antibodies. Membranes were then washed (TBS containing 0.05% Tween 20), incubated with appropriate HRP-conjugated secondary antibodies, and visualized using ECL reagent. Images were captured using an iBright CL1500 imaging system (Thermo Fisher Scientific) before densitometry was performed using ImageJ software (NIH). A complete list of primary antibodies used is provided in Supplemental Table 1.

### RNA sequencing.

Primary human Schlemm’s canal cell clone SC-68 was used for RNA sequencing. After RNA purification as described above, total RNA isolated from triplicate samples of siControl- and si*PROX1*-treated cells was provided to the NUSeq core of Northwestern University Feinberg School of Medicine for library preparation (TruSeq kit, Illumina) and sequencing on an Illumina HiSeq 4000 instrument. Reads were then aligned to the human genome, and differential expression analysis was performed using DESeq2 (85). Differentially expressed genes were then filtered by the Benjamini-Hochberg method using a false discovery threshold of 0.05, and representative genes were selected for the heatmap shown in Figure 1F.

### Solid-phase peptide synthesis.

Standard Fmoc solid-phase peptide synthesis was performed to synthesize the FLT4-binding targeting peptide (Figure 2A). Fmoc-N-amido-dPEG_24_ (Quanta Biodesign) was used in synthesis of the PG48 peptide constructs.

### Nanocarrier generation and optimization.

All polymers used for nanocarrier formulation were synthesized based on previously established procedures (35). Briefly, 40 mg of polymer, 136 μg 4OH-tamoxifen (4OHT; H6278, MilliporeSigma), and 5 μL of DiI dye (42364-100MG, Sigma-Aldrich) were dissolved in 500 μL of tetrahydrofuran (THF). This mixture was then added dropwise to 1 mL of PBS (pH 7.5) under constant rotation. Nanocarrier solution was left in a desiccator to remove THF overnight. FLT4-binding peptide (5% molar ratio) was dissolved in DMSO and incorporated with targeted nanocarriers by gentle rotation. Blank nanocarriers were synthesized by the same procedure without addition of 4OHT. Finally, the nanocarriers were purified and concentrated to a final volume of 250 μL. Drug concentration and particle size were characterized by high-performance liquid chromatography (HPLC) and dynamic light scattering.

### Quantification of 4OHT loading into nanocarriers.

Fifty-milliliter aliquots of nanocarrier solution were frozen and freeze-dried. Resulting powder was dissolved in methanol and precipitated at –20°C. Solution was then centrifuged (4,000*g*, 5 m) to separate the precipitated polymer, and supernatant was collected and analyzed. 4OHT concentration was determined using HPLC, with calibration against a 4OHT concentration series (Supplemental Figure 3, D and E) prepared in methanol. Absorbance was measured at 235 nm. Data were obtained from 3 replicate samples, and HPLC was conducted using a C18 XDB-Eclipse column (Agilent) with a static mobile phase of acetonitrile and 0.1% TFA HPLC water (85:15). 4OHT encapsulation efficiency (EE%) given as a percentage was calculated as (*M_i_* – *M_u_*)/*M_i_* × 100, where *M_i_* is the input mass of 4OHT and *M_u_* is the mass of unencapsulated 4OHT.

### Quantification of nanocarrier size, polydispersity, and ζ-potential.

To determine particle diameter size and polydispersity index (PDI), 10 mL aliquots of targeted and untargeted nanocarriers were diluted in PBS at a 10% vol/vol concentration and characterized by dynamic light scattering using a Zetasizer Nano instrument (Malvern Instruments) equipped with a 4 mW He-Ne 633 nm laser. Diameter of spherical nanocarriers and PDI were determined based on the intensity average size distributions (*n* = 3). Zeta (ζ)-potential was measured using a Zetasizer Nano device (Malvern Instruments). Average ζ-potential for each nanocarrier was determined (*n* = 3).

### Cryogenic transmission electron microscopy.

Copper grids (200 mesh) with a Lacey carbon membrane (catalog LC200-Cu-100, Electron Microscopy Sciences) were glow-discharged in a POELCO easiGlow Discharge Cleaning System (Ted Pelle, Inc.) A sample volume (4 mL) was added to the grid, blotted for 5 seconds with a blot offset of 0.5 mm, and plunged into liquid ethane within a FEI Vitrobot Mark III plunge-freezing instrument. Samples were imaged using a JEOL JEM-1230 LaB6 transmission electron microscope (JEOL USA) operating at 100 kEV.

### Small-angle x-ray scattering.

Small-angle x-ray scattering experiments were carried out at the 5-ID beamline of the DuPont-Northwestern-Dow Collaborative Access Team (DND-CAT) located at the Advanced Photon Source at Argonne National Laboratory (Lemont, Illinois, USA). Measurements were obtained using collimated x-rays with a wavelength (λ) of 1.24 Å (9 keV). Samples were prepared at a concentration of 5 mg/mL and loaded into an in-vacuum flow cell using quartz capillaries with a thickness of 1.6 mm. Scattering data were recorded over a *q*-range of 0.0015–0.08 Å^–^¹ with a sample-to-detector distance of approximately 8.5 m and an exposure time of 5 seconds. The momentum transfer, *q*, is defined as *q* = (4π/λ) sin(θ), where 2θ represents the scattering angle. Data processing, including reduction and buffer subtraction, was performed using BioXTAS RAW (86), while model fitting and analysis were conducted with SasView ((https://www.sasview.org/).

### In vitro biocompatibility of 4OHT nanocarriers.

The 3-(4,5-dimethylthiazol-2-yl)-2,5-diphenyltetrazolium bromide (MTT) assay was used to measure cell viability following incubation with nanocarriers. HUVECs, at a concentration of 5 × 10^5^ cells/mL determined by cell counting, were seeded into U-bottom 96-well plates, with 200 μL of cell suspension per well. Each well received nanocarriers in 3 replicates, prepared in PBS with final 4OHT concentrations of 0, 0.5, 1, and 2 μM. Cells were incubated with nanocarriers for 24 hours at 37°C and 5% CO_2_. MTT reagent, prepared at 5 mg/mL in PBS, was added to each well (20 μL per well), and the cells were incubated for an additional 6 hours protected from light. Afterward, the plates were centrifuged at 500*g* for 5 minutes, and the supernatant was discarded. The formazan crystals formed in the wells were dissolved with 200 μL of DMSO, and absorbance at 570 nm was measured using a SpectraMax M3 microplate reader (Molecular Devices). Cell viability was then calculated as *OD_T_*/*OD_U_* × 100%, where *OD_T_* represents optical density of the nanocarrier-treated sample and *OD_U_* corresponds to optical density of the untreated sample (*n* = 3).

### Nanocarrier uptake studies.

A total of 100,000 HUVECs or primary human Schlemm’s canal cells were plated in each well of 24-well polystyrene plates (Falcon) and allowed to adhere overnight at 37°C with 5% CO_2_. The cells were exposed to nanocarriers for 2 hours, with all incubations conducted at 37°C and 5% CO_2_. After the nanocarrier treatment, cells were harvested by mechanical scraping and then stained with fixable Zombie Aqua viability dye (BioLegend) for 20 minutes at 4°C to evaluate cytotoxicity by flow cytometry. Flow cytometry was performed using a BD LSRFortessa 6-Laser Flow Cytometer (BD Biosciences), recording 10,000 single-cell events per sample. Data were analyzed using FlowJo software (BD Biosciences). Median fluorescence intensity (MFI) was normalized by subtraction of the average MFI from untreated cells and used to assess nanocarrier uptake.

### Intracameral injection of 4OHT nanocarriers in mice.

Mice were anesthetized with isoflurane, and topical 0.5% proparacaine was provided for local analgesia. A 35-gauge beveled needle (NF35BL, World Precision Instruments) was inserted into the anterior chamber, and 3 μL nanocarrier solution was injected at a flow rate of 100 nL/min for 30 minutes using a digitally controlled Microinjection Syringe Pump (UMP3, World Precision Instruments). After injection, topical ophthalmic antibiotic (Neo-Poly-Bac, Bausch + Lomb) was applied to both eyes. Mice then received a single subcutaneous injection of meloxicam (20 mg/kg body weight) for analgesia. A second administration of topical antibiotic was provided 24 hours later.

### Tonometric IOP measurements.

Mice were anesthetized with ketamine/xylazine cocktail (112.5 mg/kg ketamine, 2.5 mg/kg xylazine), and IOP measurements were taken 5–7 minutes after anesthesia with a Tonolab rebound tonometer (iCare, Vantaa Finland). Individual Tonolab measurements represent averages of 6 individual recordings, and IOP values reported in this article are averaged from 3 measurements.

### In vivo visible-light OCT imaging and Schlemm’s canal measurement.

Visible-light optical coherence tomography (vis-OCT) uses shorter wavelengths than commonly used OCTs, thereby allowing for higher axial resolution and higher backscattering contrast in tissue. Vis-OCT was performed using a custom-built OCT system mounted on a robotic arm as previously described to image the full 360° of the aqueous humor outflow pathway (87). The system operated over a 510–610 nm spectral range, providing a theoretical axial resolution of 1.3 μm and a lateral resolution of 9.4 μm in tissue (87, 88). To achieve optimal sampling density while maintaining a compact imaging field, the lateral field of view for each volumetric scan was configured to 1.58 mm × 1.58 mm.

In vivo imaging was performed on mice under general anesthesia using an intraperitoneal injection (10 mL/kg body weight) of a ketamine/xylazine cocktail (11.45 mg/mL ketamine and 1.7 mg/mL xylazine in saline). To expose the limbal region for imaging, small relaxing incisions were made at the nasal and temporal canthi, and a circular eyelid speculum was inserted beneath the eyelids. During the imaging, a robotic arm (Meca500, Mecademic Inc.) precisely rotated the vis-OCT imaging head around the optical axis of the eye, enabling acquisition of 8 volumetric vis-OCT datasets evenly distributed around the limbus at 45° intervals (88). Each volume comprised 512 A-lines per B-scan and 512 B-scans per volume, collected using a temporal speckle-averaging acquisition protocol in which each B-scan was repeated twice per volume to improve signal-to-noise ratio and reduce speckle variation (89). The imaging beam was delivered at an A-line rate of 75 kHz with an incident optical power of 1 mW at the cornea. All quadrants of the mouse eye were imaged at the same height relative to the imaging beam to ensure uniform sampling of the circumferential Schlemm’s canal structures.

For quantitative assessment of Schlemm’s canal lumen dimensions, we analyzed 2 representative B-scans per vis-OCT volume, resulting in 16 B-scans per eye across the whole 360° imaging sequence. Each selected B-scan was chosen based on optimal visualization of the lumen and surrounding anatomical landmarks. The canal boundaries were manually segmented using MATLAB’s Volume Segmenter application, delineating the canal’s area using the paintbrush tool. The cross-sectional area of Schlemm’s canal was calculated for each segmented B-scan.

### Whole-mount imaging of mouse Schlemm’s canal.

Schlemm’s canal imaging was performed as previously described (90). Briefly, whole globes were enucleated, fixed (2% formaldehyde in PBS, pH 7.5), and dissected to remove the lens and retina. Globes were then incubated overnight in lysis/blocking buffer (5% donkey serum, 3% BSA in TBS, pH 7.5, containing 0.5% Triton X-100) before an additional overnight incubation in primary antibodies diluted in additional blocking buffer. Eyes were then washed, incubated with Alexa Fluor–conjugated secondary antibodies, and mounted for imaging. Images were captured using a Nikon A1R or Zeiss LSM 680 confocal microscope equipped with a ×20 objective. Ten- to fifteen-image *Z*-stacks were obtained, and maximum-intensity projections generated using Fiji software (91) are shown in this article. For quantification, 2–4 stacks were collected at intervals around the circumference of the eye, and total fluorescence projections were obtained using the Sum Slices function in ImageJ Fiji. Quantification of background-subtracted protein expression and canal area were obtained from these images and averaged to obtain the values reported in this article. A complete list of primary antibodies used is provided in Supplemental Table 1.

### Light and electron microscopy of mouse Schlemm’s canal.

Mice were anesthetized using ketamine/xylazine cocktail, and blood was cleared via cardiac perfusion at a flow rate of 15 mL/min (PBS containing 1 mg/mL lidocaine and 10 U/mL heparin). After clearing, 50 mL of modified Karnovsky fixative (2% paraformaldehyde and 2.5% glutaraldehyde in 0.1 M phosphate buffer, pH 7.4) was infused at the same flow rate. Upon completion of cardiac perfusion, eyes were enucleated, a small incision was made to allow fluid transfer, and eyes were submerged in additional fixative for 24 hours. Radial wedges containing trabecular meshwork and Schlemm’s canal were then collected from the nasal, inferior, superior, and temporal quadrants and prepared for ultramicrotomy and imaging as previously reported (92). Briefly, wedges were postfixed [1% OsO_4_, 1% La(NO_3_)_3_, 2 hours] before staining with 1.5% uranyl acetate. Tissues were then dehydrated, washed with propylene oxide, and embedded in Epon-Araldite using standard procedures. Gross canal morphology was analyzed in toluidine blue–stained 4 μm semithin sections by a masked investigator using ImageJ. Reported values represent averages of 7–8 sections per eye, with 1–2 sections collected from each quadrant. For electron microscopy, ultrathin sections (70–80 nm) were prepared, and imaging was performed using a JEOL JEM-1400Flash transmission electron microscope equipped with a NanoSprint43 cMOS camera (Advanced Microscopy Technologies).

### Statistics.

Statistical analysis was performed using Prism 10.6 software (GraphPad Software LLC). Tests used to obtain reported *P* values are described in the figure legends. Figures were prepared using Prism 10.6, Illustrator 29.8.1, and InDesign 20.5 software (Adobe Inc.). Image analysis and quantification were performed using ImageJ Fiji (92). Except where noted, all error bars shown in figures indicate SEM.

### Study approval.

Animal experiments were approved by the Animal Care and Use Committee at Northwestern University under animal protocols IS00020857 and IS00022641 and complied with the Association for Research in Vision and Ophthalmology guidelines for care and use of vertebrate research subjects in ophthalmology research.

### Data availability.

Raw transcriptomics data are available in the NCBI’s Gene Expression Omnibus (GEO) database using accession number GSE310660. A table of all differentially expressed genes identified after si*PROX1* treatment and used to generate Figure 1F is provided as Supplemental Dataset 1. Raw data underlying other figures are available upon reasonable request.

## Author contributions

BRT, MJ, and EAS conceived of the study. SLO, BRT, MJ, EAS, HG, HFZ, ZY, and HLL designed experiments and analyzed results. SLO, MPV, HLL, NCM, PL, HK, SA, ZY, HJL, and BRT conducted experiments. SLO, BRT, MJ, and EAS wrote the manuscript. All authors contributed to editing the manuscript and validation of the results.

## Conflict of interest

EAS, MJ, BRT, SLO, and NCM have submitted a provisional patent application related to therapeutic targeting of PROX1 in ocular hypertension and glaucoma. EAS, MJ, and MPV have applied for patent 20240307358 related to PEG-b-PPS nanocarrier–based glaucoma therapies, and EAS holds and has applied for US patents 12194084, 12162997, 20230263729, 11202823, 20200383917related to PEG-b-PPS–based therapies for other diseases. In addition, EAS is the CEO and founder of SNC Therapeutics Inc., a startup company focused on developing a gene delivery vehicle based on the PEG-PPS platform. HFZ holds US patents 12623407, 10750943, 9962075, 20250031969, and 20240361665 on visible-light OCT–related technologies and is a founder of Opticent Health.

## Funding support

This work is the result of NIH funding, in whole or in part, and is subject to the NIH Public Access Policy. Through acceptance of this federal funding, the NIH has been given a right to make the work publicly available in PubMed Central.

- National Institutes of Health (NIH) R01 EY033813 (to MJ and EAS).
- A Northwestern University Catalyst Grant (to MJ and BRT).
- Christina Enroth-Cugell Graduate Research Award (to SLO).
- NIH R01 R01EY032609 (to BRT).
- Brightfocus Foundation grant M2021018N (to BRT).
- National Cancer Institute CCSG P30 CA060553, supporting the Center for Advanced Microscopy of the Feinberg School of Medicine, where imaging was performed.
- NIH grant S10 OD028571, supporting the Boston University Transmission Electron Microscopy Core, where imaging was performed.
- George M. O’Brien kidney core grant P30 DK114857.
- Research to Prevent Blindness challenge grant to the Feinberg School of Medicine Department of Ophthalmology.

## Supplementary Material

Supplemental data

Supplemental data set 1

Unedited blot and gel images

Supporting data values

## Figures and Tables

**Table 1:**
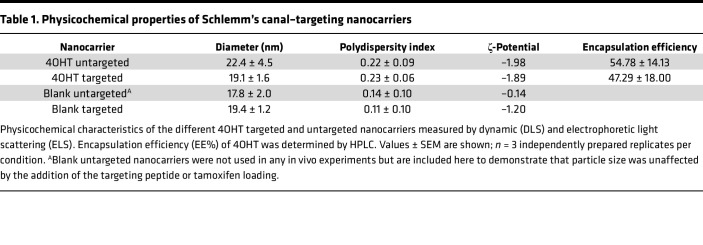
Physicochemical properties of Schlemm’s canal–targeting nanocarriers

## Version 1 05/05/2026

In-Press Preview

## Version 2 06/22/2026

Electronic publication

## Version 3 06/23/2026

Typographical errors in abstract corrected

## References

[1] Quigley HA, Broman AT (2006). The number of people with glaucoma worldwide in 2010 and 2020. Br J Ophthalmol.

[2] Kass MA (2002). The Ocular Hypertension Treatment Study: a randomized trial determines that topical ocular hypotensive medication delays or prevents the onset of primary open-angle glaucoma. Arch Ophthalmol.

[3] De Moraes CG (2012). Effect of treatment on the rate of visual field change in the ocular hypertension treatment study observation group. Invest Ophthalmol Vis Sci.

[4] Ederer F (1994). The Advanced Glaucoma Intervention Study (AGIS): 1. Study design and methods and baseline characteristics of study patients. Control Clin Trials.

[5] Sheybani A (2020). Open-angle glaucoma: burden of illness, current therapies, and the management of nocturnal IOP variation. Ophthalmol Ther.

[6] Grant WM (1951). Clinical measurements of aqueous outflow. Am J Ophthalmol.

[7] Leber T (1873). Studien über den flüssigkeitswechsel im auge. Albrecht von Graefes Archiv für Ophthalmologie.

[8] Johnson M (1992). Modulation of outflow resistance by the pores of the inner wall endothelium. Invest Ophthalmol Vis Sci.

[9] Allingham RR (1992). The relationship between pore density and outflow facility in human eyes. Invest Ophthalmol Vis Sci.

[10] Johnson M (2002). The pore density in the inner wall endothelium of Schlemm’s canal of glaucomatous eyes. Invest Ophthalmol Vis Sci.

[11] Overby DR (2014). Altered mechanobiology of Schlemm’s canal endothelial cells in glaucoma. Proc Natl Acad Sci U S A.

[12] Vahabikashi A (2019). Increased stiffness and flow resistance of the inner wall of Schlemm’s canal in glaucomatous human eyes. Proc Natl Acad Sci U S A.

[13] Zhou EH (2012). Mechanical responsiveness of the endothelial cell of Schlemm’s canal: scope, variability and its potential role in controlling aqueous humour outflow. J R Soc Interface.

[14] Raviola G, Raviola E (1981). Paracellular route of aqueous outflow in the trabecular meshwork and canal of Schlemm. A freeze-fracture study of the endothelial junctions in the sclerocorneal angel of the macaque monkey eye. Invest Ophthalmol Vis Sci.

[15] Ramos RF (2007). Schlemm’s canal endothelia, lymphatic, or blood vasculature?. J Glaucoma.

[16] Pawlak JB, Caron KM (2020). Lymphatic programing and specialization in hybrid vessels. Front Physiol.

[17] Kenig-Kozlovsky Y (2017). Ascending vasa recta are angiopoietin/tie2-dependent lymphatic-like vessels. J Am Soc Nephrol.

[18] Hong SP (2023). Three-dimensional morphologic and molecular atlases of nasal vasculature. Nat Cardiovasc Res.

[19] Pawlak JB (2019). Lymphatic mimicry in maternal endothelial cells promotes placental spiral artery remodeling. J Clin Invest.

[20] Schnabellehner S (2024). Penile cavernous sinusoids are Prox1-positive hybrid vessels. Vasc Biol.

[21] Kizhatil K (2014). Schlemm’s canal is a unique vessel with a combination of blood vascular and lymphatic phenotypes that forms by a novel developmental process. PLoS Biol.

[22] O’Morchoe CC, O’Morchoe PJ (1987). Differences in lymphatic and blood capillary permeability: ultrastructural-functional correlations. Lymphology.

[23] Oliver G (2022). Lymphatic endothelial cell fate specification in the mammalian embryo: an historical perspective. Dev Biol.

[24] Johnson M (2006). What controls aqueous humour outflow resistance?. Exp Eye Res.

[25] Aspelund A (2014). The Schlemm’s canal is a VEGF-C/VEGFR-3-responsive lymphatic-like vessel. J Clin Invest.

[26] Van Der Merwe EL, Kidson SH (2014). The three-dimensional organisation of the post-trabecular aqueous outflow pathway and limbal vasculature in the mouse. Exp Eye Res.

[27] Thomson BR (2021). Cellular crosstalk regulates the aqueous humor outflow pathway and provides new targets for glaucoma therapies. Nat Commun.

[28] Kim J (2017). Impaired angiopoietin/Tie2 signaling compromises Schlemm’s canal integrity and induces glaucoma. J Clin Invest.

[29] Park DY (2014). Lymphatic regulator PROX1 determines Schlemm’s canal integrity and identity. J Clin Invest.

[30] Reina-Torres E (2017). VEGF as a paracrine regulator of conventional outflow facility. Invest Ophthalmol Vis Sci.

[31] Thomson BR (2020). Angiopoietin-1 knockout mice as a genetic model of open-angle glaucoma. Transl Vis Sci Technol.

[32] Thackaberry EA (2019). Rapid development of glaucoma via ITV nonselective ANGPT 1/2 antibody: a potential role for ANGPT/TIE2 signaling in primate aqueous humor outflow. Invest Ophthalmol Vis Sci.

[33] Schwakopf J (2024). Schlemm’s canal-selective Tie2/TEK knockdown induces sustained ocular hypertension in adult mice. Exp Eye Res.

[34] Stack T (2018). Modulation of Schlemm’s canal endothelial cell stiffness via latrunculin loaded block copolymer micelles. J Biomed Mater Res A.

[35] Stack T (2020). Targeted delivery of cell softening micelles to Schlemm’s canal endothelial cells for treatment of glaucoma. Small.

[36] Vincent MP (2021). Surface engineering of FLT4-targeted nanocarriers enhances cell-softening glaucoma therapy. ACS Appl Mater Interfaces.

[37] Rashbrook VS (2022). Cre toxicity in mouse models of cardiovascular physiology and disease. Nat Cardiovasc Res.

[38] Van Zyl T (2020). Cell atlas of aqueous humor outflow pathways in eyes of humans and four model species provides insight into glaucoma pathogenesis. Proc Natl Acad Sci U S A.

[39] Balasubramanian R (2024). Transcriptomic profiling of Schlemm’s canal cells reveals a lymphatic-biased identity and three major cell states. Elife.

[40] Van Zyl T (2022). Cell atlas of the human ocular anterior segment: tissue-specific and shared cell types. Proc Natl Acad Sci U S A.

[41] Patel G (2020). Molecular taxonomy of human ocular outflow tissues defined by single-cell transcriptomics. Proc Natl Acad Sci U S A.

[42] Birke K (2010). Expression of podoplanin and other lymphatic markers in the human anterior eye segment. Invest Ophthalmol Vis Sci.

[43] Johnson NC (2008). Lymphatic endothelial cell identity is reversible and its maintenance requires Prox1 activity. Genes Dev.

[44] Srinivasan RS (2014). The Prox1-Vegfr3 feedback loop maintains the identity and the number of lymphatic endothelial cell progenitors. Genes Dev.

[45] Harvey NL (2005). Lymphatic vascular defects promoted by Prox1 haploinsufficiency cause adult-onset obesity. Nat Genet.

[46] Niec RE (2022). Lymphatics act as a signaling hub to regulate intestinal stem cell activity. Cell Stem Cell.

[47] Gaasterland D (1978). Studies of aqueous humour dynamics in man. VI. Effect of age upon parameters of intraocular pressure in normal human eyes. Exp Eye Res.

[48] Brauch H (2009). Pharmacogenomics of tamoxifen therapy. Clin Chem.

[49] Ma W (2021). Mitochondrial respiration controls the Prox1-Vegfr3 feedback loop during lymphatic endothelial cell fate specification and maintenance. Sci Adv.

[50] Wigle JT, Oliver G (1999). Prox1 function is required for the development of the murine lymphatic system. Cell.

[51] Petrova TV (2002). Lymphatic endothelial reprogramming of vascular endothelial cells by the Prox-1 homeobox transcription factor. EMBO J.

[52] Overby DR (2014). Ultrastructural changes associated with dexamethasone-induced ocular hypertension in mice. Invest Ophthalmol Vis Sci.

[53] Last JA (2011). Elastic modulus determination of normal and glaucomatous human trabecular meshwork. Invest Ophthalmol Vis Sci.

[54] Zhang RZ (2007). Length-tension relationships of small arteries, veins, and lymphatics from the rat mesenteric microcirculation. Am J Physiol Heart Circ Physiol.

[55] Thomson BR (2019). Targeting the vascular-specific phosphatase PTPRB protects against retinal ganglion cell loss in a pre-clinical model of glaucoma. Elife.

[56] Mishra S (2025). Inhibition of VE-PTP rejuvenates Schlemm’s canal in aged mice and acts via Tie2. PLoS One.

[57] Li G (2020). A small molecule inhibitor of VE-PTP activates Tie2 in Schlemm’s canal increasing outflow facility and reducing intraocular pressure. Invest Ophthalmol Vis Sci.

[58] Rämö JT (2025). Rare genetic variation in PTPRB is associated with central serous chorioretinopathy, varicose veins and glaucoma. Nat Commun.

[59] Souma T (2018). Context-dependent functions of angiopoietin 2 are determined by the endothelial phosphatase VEPTP. Proc Natl Acad Sci U S A.

[60] Thomson BR (2014). A lymphatic defect causes ocular hypertension and glaucoma in mice. J Clin Invest.

[61] Biswas S, Wan KH (2019). Review of rodent hypertensive glaucoma models. Acta Ophthalmol.

[62] Turner AJ (2017). DBA/2J mouse model for experimental glaucoma: pitfalls and problems. Clin Exp Ophthalmol.

[63] Zhang X (2024). Intraocular pressure across the lifespan of tg-myocy437h mice. Exp Eye Res.

[64] Bahranifard MR (2025). Magnetically steered cell therapy for reduction of intraocular pressure as a treatment strategy for open-angle glaucoma. Elife.

[65] Tolman NG (2025). Absence of glaucoma in Tg-MYOCY437H mice of diverse genetic backgrounds. Invest Ophthalmol Vis Sci.

[66] Jonas JB (2014). Ocular hypertension: general characteristics and estimated cerebrospinal fluid pressure. The Beijing Eye Study 2011. PLoS One.

[67] Wang YX (2018). Intraocular pressure and its normal range adjusted for ocular and systemic parameters. The Beijing Eye Study 2011. PLoS One.

[68] Yi S (2016). Tailoring nanostructure morphology for enhanced targeting of dendritic cells in atherosclerosis. ACS Nano.

[69] Vincent MP (2021). Surface chemistry-mediated modulation of adsorbed albumin folding state specifies nanocarrier clearance by distinct macrophage subsets. Nat Commun.

[70] Vincent MP (2021). The combination of morphology and surface chemistry defines the immunological identity of nanocarriers in human blood. Adv Ther (Weinh).

[71] Russell S (2017). Efficacy and safety of voretigene neparvovec (AAV2-hRPE65v2) in patients with RPE65-mediated inherited retinal dystrophy: a randomised, controlled, open-label, phase 3 trial. Lancet.

[72] Cukras C (2018). Retinal AAV8-RS1 gene therapy for X-linked retinoschisis: initial findings from a Phase I/IIa trial by intravitreal delivery. Mol Ther.

[73] Lopez J (2023). Subretinal deposits in young patients treated with voretigene neparvovec-rzyl for RPE65-mediated retinal dystrophy. Br J Ophthalmol.

[74] Gange WS (2022). Perifoveal chorioretinal atrophy after subretinal voretigene neparvovec-rzyl for RPE65-mediated Leber congenital amaurosis. Ophthalmol Retina.

[75] Wiley LA (2023). The degree of adeno-associated virus-induced retinal inflammation varies based on serotype and route of delivery: intravitreal, subretinal, or suprachoroidal. Hum Gene Ther.

[76] Kim H (2026). Filomicelle-embedded composite hydrogels for localized gelation within the anterior chamber of the eye. Small.

[77] Yi S (2019). Surface engineered polymersomes for enhanced modulation of dendritic cells during cardiovascular immunotherapy. Adv Funct Mater.

[78] Burke JA (2022). Subcutaneous nanotherapy repurposes the immunosuppressive mechanism of rapamycin to enhance allogeneic islet graft viability. Nat Nanotechnol.

[79] Vasdekis AE (2012). Precision intracellular delivery based on optofluidic polymersome rupture. ACS Nano.

[80] Kaipa BR (2025). Impaired axonal transport contributes to neurodegeneration in a Cre-inducible mouse model of myocilin-associated glaucoma. JCI Insight.

[81] Kaser-Eichberger A (2015). Topography of lymphatic markers in human iris and ciliary body. Invest Ophthalmol Vis Sci.

[82] Muzumdar MD (2007). A global double-fluorescent Cre reporter mouse. Genesis.

[83] Sörensen I (2009). DLL1-mediated Notch activation regulates endothelial identity in mouse fetal arteries. Blood.

[84] Donnan MD (2023). Formation of the glomerular microvasculature is regulated by VEGFR-3. Am J Physiol Renal Physiol.

[85] Love MI (2014). Moderated estimation of fold change and dispersion for RNA-seq data with DESeq2. Genome Biol.

[86] Hopkins JB (2017). BioXTAS RAW: improvements to a free open-source program for small-angle X-ray scattering data reduction and analysis. J Appl Crystallogr.

[87] Zhang X (2020). In vivo imaging of Schlemm’s canal and limbal vascular network in mouse using visible-light OCT. Invest Ophthalmol Vis Sci.

[88] Fang R (2025). Robotic optical coherence tomography with expanded three-dimensional field-of-view using point-cloud-based volumetric montaging. IEEE Trans Med Imaging.

[89] Zhang P (2019). Temporal speckle-averaging of optical coherence tomography volumes for *in-vivo* cellular resolution neuronal and vascular retinal imaging. Neurophotonics.

[91] Schindelin J (2012). Fiji: an open-source platform for biological-image analysis. Nat Methods.

[92] Li HL (2025). Endothelial glycocalyx in different flow regions of the trabecular outflow pathway in bovine eyes. Front Cell Dev Biol.

