# Supplementary material for: PROX1 loss in adult mouse Schlemm’s canal causes permanent ocular hypertension
Source: JCI Insight. 2026 May 5;11(12):e203711. doi: 10.1172/jci.insight.203711 (PMC13313505; doi:10.1172/jci.insight.203711)
Supplement: Supplemental data [file jciinsight-11-203711-s324.pdf]

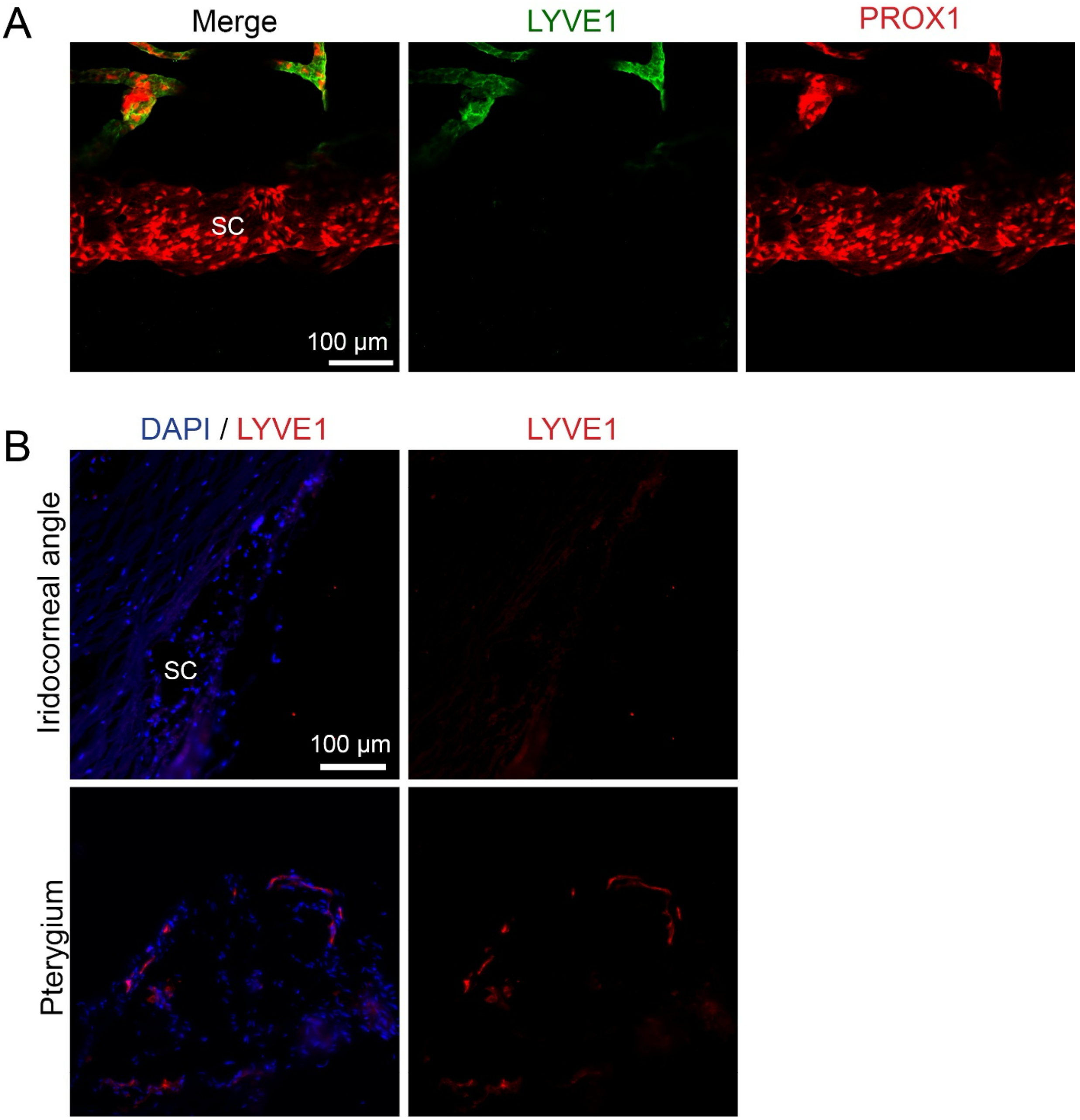

Supplemental Figure 1. The lymphatic protein LYVE1 is not expressed in mouse or human Schlemm's canal (SC). (A) Whole mount immunostaining of mouse SC, and (B) cryopreserved cross-sections of human SC revealed no expression of the common lymphatic marker LYVE1, although robust expression was observed in adjacent lymphatic vessels of the mouse eye and in lymphatic vessels of human pterygium tissue used as a positive control.

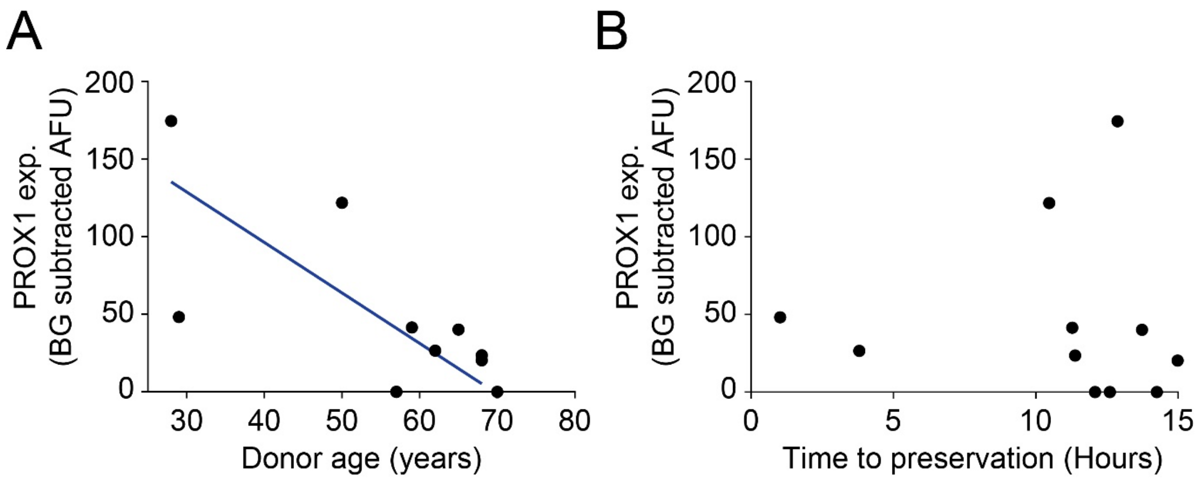

Supplemental Figure 2. PROX1 expression was negatively correlated with age in human corneal rim cryosections. Results from multivariate regression of nuclear PROX1 immunostaining intensity against donor age and time to preservation (Full model, p=0.028, adjusted R^2 = 0.4887, n = 10 donors) (A) PROX1 staining intensity within ERG-positive Schlemm's canal endothelial nuclei was negatively correlated with age (age, p<0.01) in human corneal rim sections. (B) PROX1 staining intensity did not decrease with time from death to tissue preservation for eyes preserved in less than 15 hours of death (time to preservation, p>0.6).

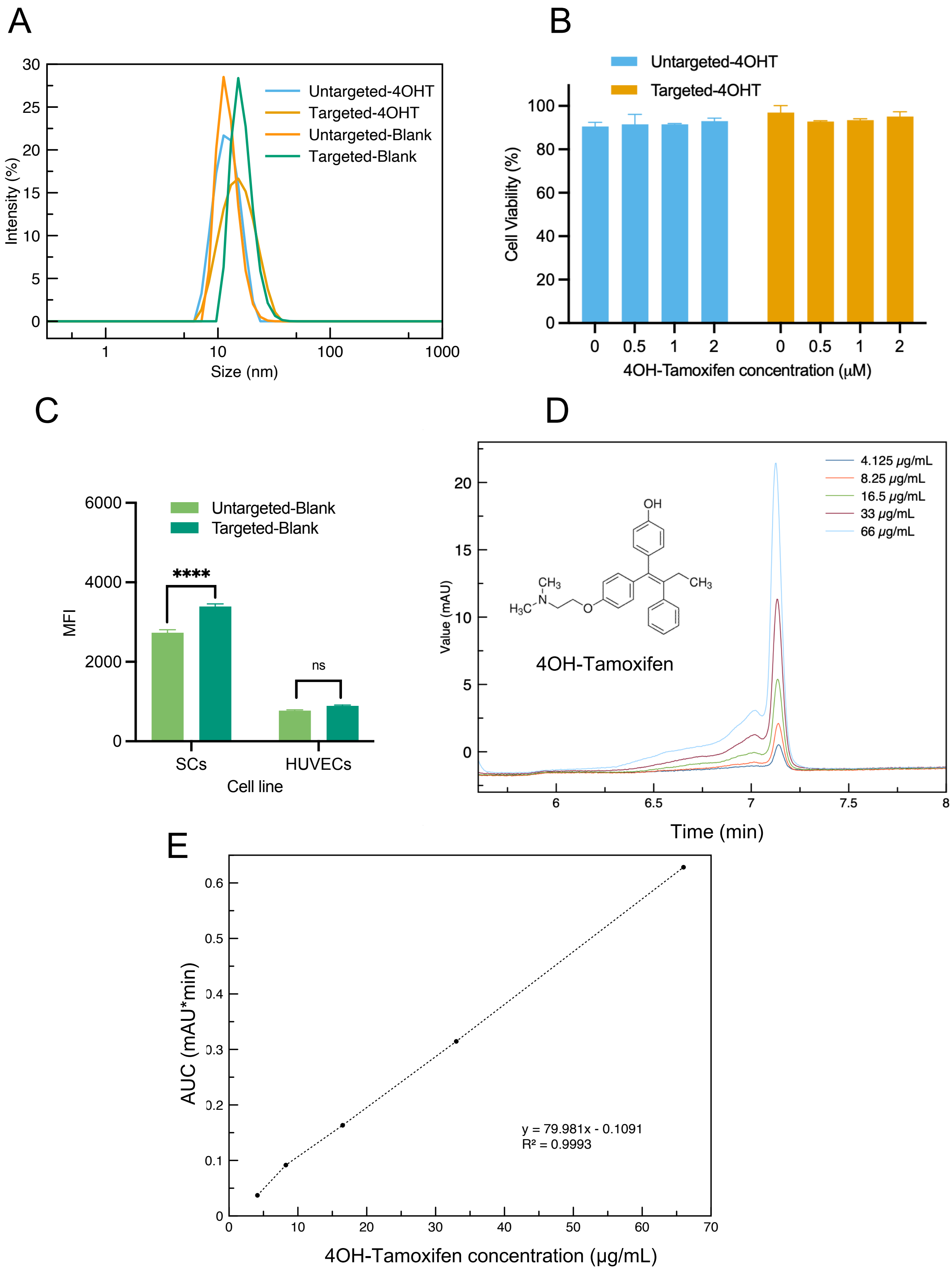

A
Intensity (%)
Size (nm)
Untargeted-4OHT
Targeted-4OHT
Untargeted-Blank
Targeted-Blank
B
Untargeted-4OHT
Targeted-4OHT
Cell Viability (%)
4OH-Tamoxifen concentration (μM)
C
Untargeted-Blank
Targeted-Blank
MFI
****
ns
SCs
HUVECs
Cell line
D
4.125 μg/mL
8.25 μg/mL
16.5 μg/mL
33 μg/mL
66 μg/mL
Value (mAU)
OH
CH3
H3C
N
O
CH3
4OH-Tamoxifen
Time (min)
E
AUC (mAU*min)
y = 79.981x - 0.1091
R² = 0.9993
4OH-Tamoxifen concentration (μg/mL)

Supplemental Figure 3. (A) DLS size distribution of 4 different targeted or untargeted samples containing 4OH-Tamoxifen fabricated with cosolvent evaporation. (B) MTT assay revealed that HUVEC cell viability was not affected by a 4h incubation with either 4OH-Tamoxifen targeted or untargeted nanocarriers. (C) Decoration with a VEGFC-derived targeting peptide increased uptake of DiI-labeled blank nanocarriers by primary human Schlemm's canal endothelial cells but not HUVECs. The mean fluorescence intensity (MFI) $\pm$ s.e.m. (n=4) is displayed. Significance was determined by ANOVA followed by Tukey's multiple comparisons test, 5% significance level was used for all statistical tests. ****$p<0.0001$. (D) HPLC chromatograms of 4OH-Tamoxifen that referenced a reproducible 4OHT concentration series for standard curve calibration of High-Performance Liquid Chromatography (HPLC) measurements. A linear regression model fitted to the data: $y = 79.981x + 0.1091$, $r^2 = 0.9993$ to determine the concentration of 4OH-Tamoxifen of the samples. (E) Standard curve of serially diluted free form 4OHT. A C18 XDB-Eclipse column (Agilent) was used with a static mobile phase of acetonitrile and 0.1% (v/v%) TFA water (85:15).

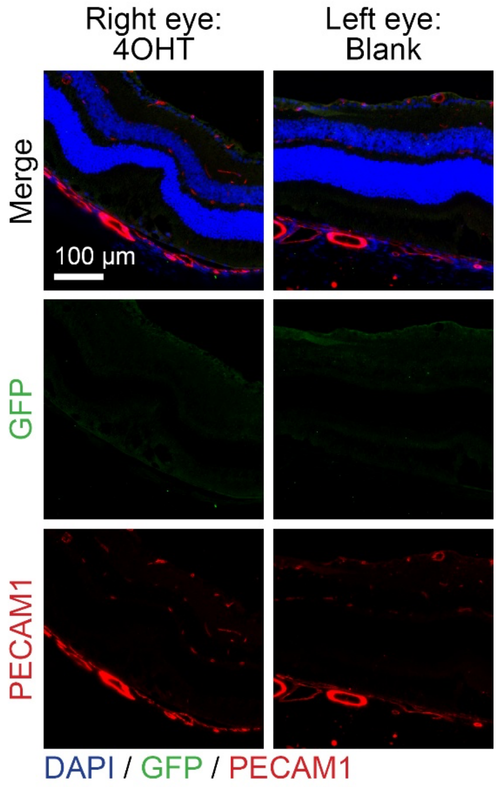

Supplemental Figure 4. No recombination was observed in retinal or choroidal capillaries of eye cryosections prepared from *Rosa26*^{mTmG}; *Cdh5*-CreERT2 mice 7 days after receiving two intracameral injections of targeted 4OHT-loaded or identical, targeted blank nanocarriers.

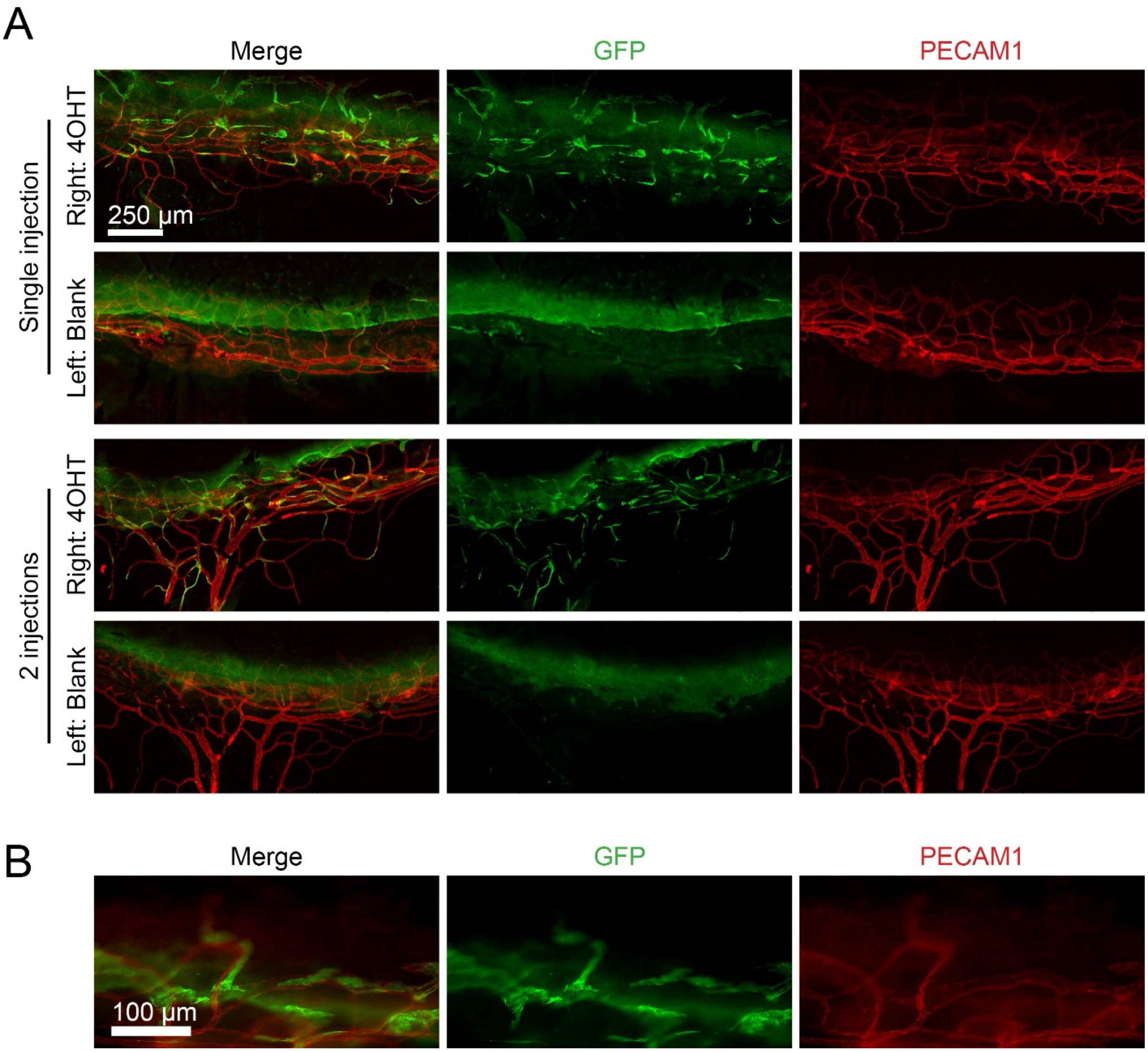

Supplemental Figure 5. Sporadic recombination was observed in distal outflow and limbal blood vessels of eyes treated with 4-OH-tamoxifen loaded, Schlemm's canal targeted nanocarriers. (A) 7 days after intracameral nanocarrier delivery, low levels of sporadic cre-mediated recombination (determined by GFP fluorescence) was observed in *Rosa26*^{mTmG}; *Cdh5*-CreERT2 mice (GFP expression) treated with 4OHT nanocarriers. Similar levels of recombination were seen in eyes receiving one or two nanocarrier infusions, while very few recombined cells were observed in contralateral control eyes that received matching injections of empty control nanocarriers. (B) Recombination was also observed in endothelial cells of limbal lymphatic capillaries of some eyes receiving targeted 4OHT nanocarriers, consistent with their known expression of FLT4.

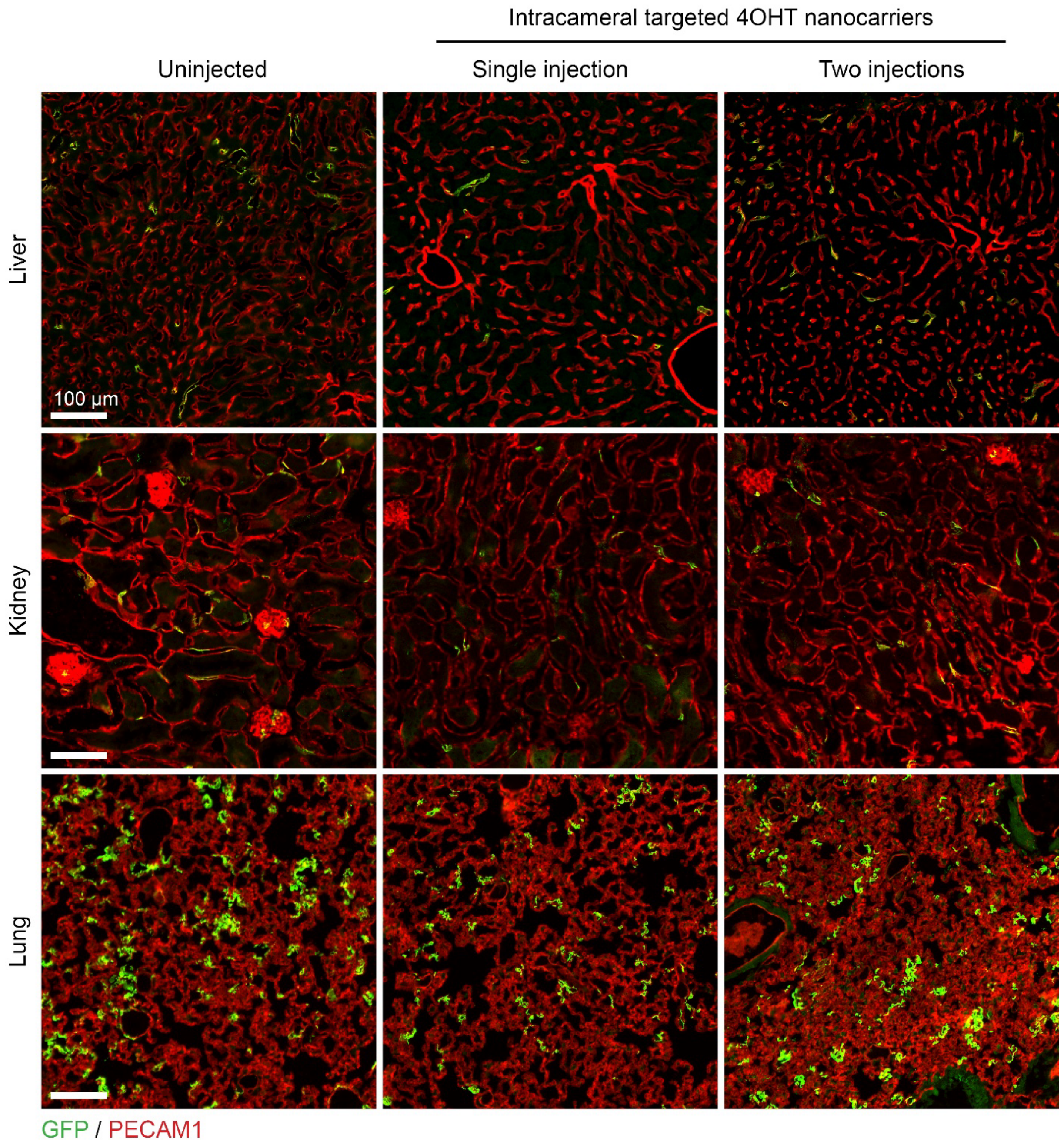

Supplemental Figure 6. Outside of the eye, no increase in cre-mediated recombination was observed following intracameral injection with 4OHT loaded nanocarriers. Cryosections were prepared from liver, kidney and lung tissues of *Rosa26*^{mTmG}; *Cdh5*-CreERT2 mice seven days after intracameral injection with 4OHT-loaded Schlemm's canal targeting nanocarriers, and cre mediated recombination was analyzed by GFP fluorescence. While some recombination was observed in endothelial cells of all organs, number of recombined endothelial cells was similar in untreated and nanocarrier-treated mice, suggesting this was due to cre leakiness and not nanocarrier activity.

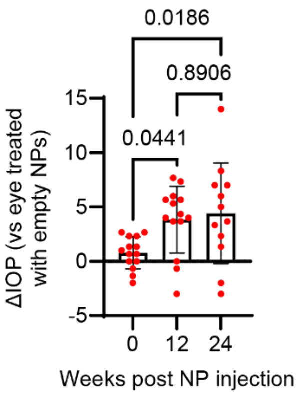

Supplemental Figure 7. Prolonged IOP elevation in a second cohort of *Prox1*$^{flox/flox}$; *Cdh5*-CreERT2 mice after 4-OH tamoxifen nanocarrier treatment. IOP elevation was maintained in Cre-positive mice until animals were euthanized 24 weeks after nanocarrier-mediated PROX1 deletion. Error bars indicate ±SEM, reported p values were obtained using a 1-way ANOVA followed by Tukey's multiple comparison test. n = 14 mice.

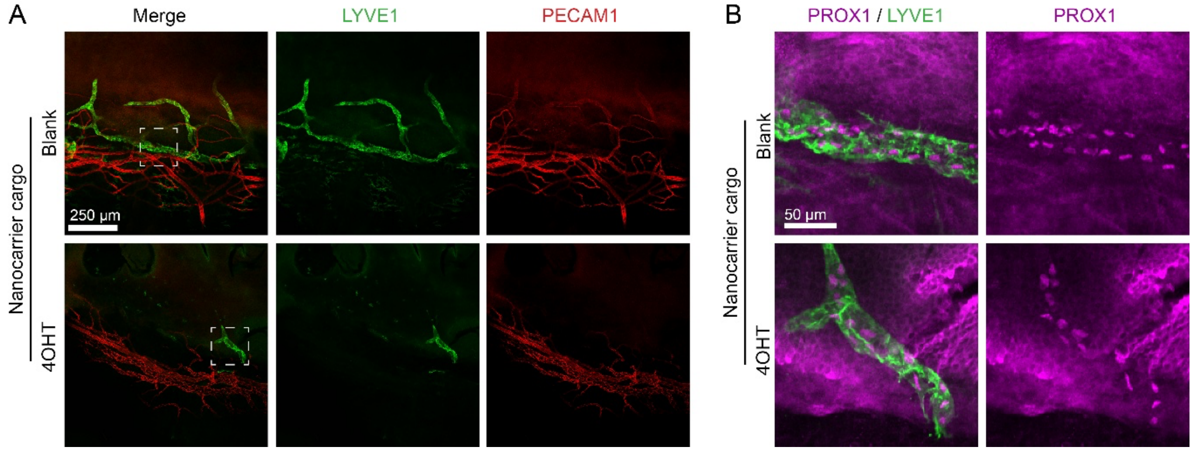

Supplemental Figure 8. Degeneration of limbal lymphatic vessels was observed in *Prox1*$^{flox/flox}$; *Cdh5*-CreERT2 eyes following 4OH-tamoxifen nanocarrier induction. (A) Compared with the organized pattern of limbal lymphatic capillaries in *Prox1*$^{flox/flox}$; *Cdh5*-CreERT2 eyes treated with blank control nanocarriers, confocal microscopy at the level of the limbal vascular network revealed degeneration of limbal lymphatics in contralateral eyes receiving 4OHT nanocarriers. (B) Normal PROX1 expression was observed in remaining lymphatic endothelial cells of 4OHT-treated eyes. Dashed white square in (A) indicates regions of detail highlighted in (B).

**Supplemental Table S1. Primary antibodies used**	
Mouse anti PROX1	Developmental Studies Hybridoma Bank, Iowa City, IA USA, 1A6
Goat anti LYVE1	R&D systems AF2125
Rabbit anti ERG	Abcam, Waltham MA, USA, ab92513
Rabbit anti human GAPDH	Cell Signaling, Danvers MA, #2118
Goat anti human PROX1	R&D systems AF2727
Rabbit anti mouse PROX1	Sigma-Aldrich-Chemicon, AB5475
Goat anti mouse FLT4	R&D Systems AF349
Rat anti mouse PECAM1	BD Biosciences AB397095
Goat anti human PECAM1	R&D Systems AF3628
