# Supplementary figures and images for: PROX1 loss in adult mouse Schlemm’s canal causes permanent ocular hypertension
Source: JCI Insight. 2026 May 5;11(12):e203711. doi: 10.1172/jci.insight.203711 (PMC13313505; doi:10.1172/jci.insight.203711)

FLT4

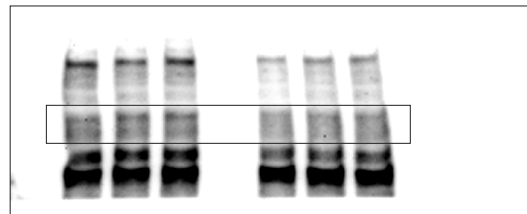

- 150kDa

GAPDH

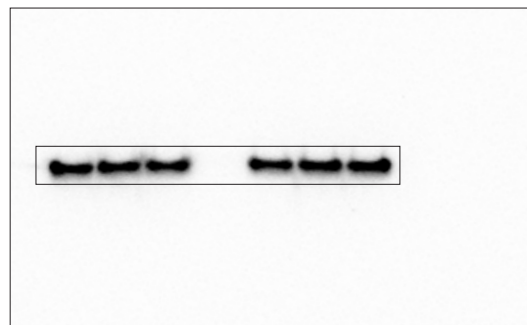

- 50kDa

siControl

siPROX1

Supplement: Unedited blot and gel images [file jciinsight-11-203711-s326.pdf]
